# Targeted CRISPR/Cas9 Lipid Nanoparticles Elicits Therapeutic Genome Editing in Head and Neck Cancer

**DOI:** 10.1002/advs.202411032

**Published:** 2024-12-30

**Authors:** Razan Masarwy, Dor Breier, Lior Stotsky‐Oterin, Nitay Ad‐El, Shahd Qassem, Gonna Somu Naidu, Anjaiah Aitha, Assaf Ezra, Meir Goldsmith, Inbal Hazan‐Halevy, Dan Peer

**Affiliations:** ^1^ Laboratory of Precision Nanomedicine The Shmunis School of Biomedicine and Cancer Research George S. Wise Faculty of Life Sciences Tel Aviv University Tel Aviv 69978 Israel; ^2^ Department of Materials Sciences and Engineering Iby and Aladar Fleischman Faculty of Engineering Tel Aviv University Tel Aviv 69978 Israel; ^3^ Center for Nanoscience and Nanotechnology Tel Aviv University Tel Aviv 69978 Israel; ^4^ Cancer Biology Research Center Tel Aviv University Tel Aviv 69978 Israel; ^5^ School of Medicine Tel Aviv University Tel Aviv 69978 Israel

**Keywords:** CRISPR‐Cas9, Genetic Medicines, Head and neck cancer, Lipid nanoparticles, mRNA, SOX2

## Abstract

Squamous cell carcinomas of the head and neck (HNSCC) originate in the upper aerodigestive tract, including the oral cavity, pharynx, and larynx. Current treatments of locally advanced HNSCC often lead to high treatment failure, and disease recurrence, resulting in poor survival rates. Advances in mRNA technologies and lipid nanoparticle (LNP) delivery systems led to several clinical trials involving LNP‐CRISPR‐Cas9 mRNA‐based therapeutics. Despite these advances, achieving cell‐type‐specific extrahepatic mRNA delivery is still challenging. This study introduces a safe and effective intratumoral EGFR‐targeted CRISPR‐LNP delivery strategy for knocking out SOX2, which is a cancer‐specific gene. To assess their therapeutic potential, it is shown that LNPs made from ionizable lipids with helper lipids co‐encapsulating Cas9 mRNA and sgRNA targeting SOX2 (sgSOX2), lead to a ≈60% reduction in HNSCC cell viability in vitro. Next, using a xenograft HNSCC mouse model, targeted delivery of 𝜶EGFR‐ CRISPR‐sgSOX2‐LNPs to HNSCC cells resulted in a 90% inhibition of tumor growth and a 90% increase in survival for > 84 days, with tumor disappearance observed in 50% of the mice. These findings emphasize the potential of targeted mRNA‐Cas9‐LNPs in clinically accessible solid tumors, specifically in reaching tumor cells and inducing persistent therapeutic responses in tumors with high‐recurrence rates like HNSCC.

## Graphical abstract

αEGFR‐t‐SOX2‐cLNPs achieved specific delivery to tumor cells, resulting in 90% tumor growth inhibition of HNSCC‐bearing mice. This response is sustained for 12 weeks, with 50% of the mice showing tumor disappearance. These findings emphasize the potential of targeted mRNA‐Cas9‐LNPs in clinically accessible solid tumors, specifically in reaching tumor cells and inducing persistent therapeutic responses in cancers with high recurrence rates such as HNSCC.

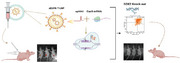

## 1 Introduction

Head and neck squamous cell carcinomas (HNSCCs) develop from the mucosal epithelium in the oral cavity, pharynx, and larynx.^[^ ^1^ ^]^ It is the sixth most common cancer worldwide, resulting in ≈600000 new cases and 300000 deaths recorded every year.^[^ ^2^ ^]^ The standard of care for locoregionally advanced HNSCC patients includes either surgical resection combined with adjuvant therapy or definitive chemoradiotherapy.^[^ ^3^, ^4^ ^]^ Yet, despite this multimodal therapeutic approach, HNSCC patients suffer from high rates of treatment failure, treatment‐related toxicities, and disease recurrence which together are responsible for poor survival outcomes.^[^ ^1^ ^]^

Lipid nanoparticles (LNPs) are the most advanced nonviral strategy for efficient in vivo RNA encapsulation and delivery.^[^ ^5^, ^6^ ^]^ In recent years several projects were started, aiming to harness this platform for CRISPR‐Cas9 therapeutics, including oncology, supported by numerous in vivo studies demonstrating the potential of mRNA‐Cas9‐LNPs in cancer treatment.^[^ ^7^, ^8^, ^9^ ^]^ However, the translation of this technology into clinical applications faces various challenges,^[^ ^10^ ^]^ including challenges inherent to the CRISPR‐Cas9 system itself, the necessity for achieving cell‐type‐specific and extrahepatic delivery of the mRNA‐Cas9‐LNPs and the unique characteristics of tumors.

The first tumor‐related challenge is its limitless replicative potential. Due to the highly proliferative nature of the tumor, editing a small number of cells may not suffice to reverse the progression of the disease, therefore we need sufficient editing percentages.^[^ ^11^, ^12^ ^]^ Second, the dense microenvironment of solid tumors serves as a barrier, impeding CRISPR‐Cas9 cargo uptake and restricting access to the number of cells needed to counter their replicative potential.^[^ ^9^, ^12^ ^]^ Therefore, in HNSCC, systemic administration of CRISPR‐loaded LNPs may yield poor tumor penetration and undesired liver accumulation.^[^ ^7^, ^11^ ^]^ However, in HNSCC many lesions are visible and palpable, making them suitable for intratumoral therapy. Even though intratumoral LNP delivery holds the potential to reach more cells compared to systemic injections it still doesn't ensure exclusive expression in cancer cells.^[^ ^7^, ^13^ ^]^ As such, active LNP targeting, achieved by attaching an antibody that binds a specific receptor highly expressed on HNSCC cells, is essential to enhance specific uptake and safety.^[^ ^14^, ^15^, ^16^, ^17^ ^]^ Due to its overexpression, the epidermal growth factor receptor (EGFR) is a promising target moiety for LNP delivery to HNSCC cells.^[^ ^18^, ^19^ ^]^ Additionally, cetuximab, an FDA‐approved EGFR‐targeting therapy since 2006, is used alone or with standard treatments for HNSCC patients not responding to platinum‐based therapy or with recurrent/metastatic disease.^[^ ^20^, ^21^ ^]^

Regarding CRISPR‐Cas9 challenges, they mainly stem from both on‐target and off‐target activity. Induction of double‐stranded breaks can lead to unintended large deletions and chromosomal aberrations, potentially causing another cancer.^[^ ^22^ ^]^ Targeting tumor‐specific genes is crucial to minimize this risk. Additionally, off‐target effects may lead to unwanted gene disruption or cellular damage, necessitating precise delivery to cancerous cells. Choosing a tumor‐specific target gene, exclusively expressed in cancerous cells and vital for their survival, can mitigate off‐tumor activity risks specifically arising from the knockout of the target.^[^ ^22^ ^]^ SOX2 is a critical transcription factor for embryogenesis and stem cell pluripotency. In normal adult tissues, SOX2 is primarily expressed in stem and progenitor cells within epithelial tissues like the stomach, cervix, testes, lens, and glands (e.g., salivary, mammary, and respiratory glands). It helps regulate tissue homeostasis, repair, and regeneration, maintaining a balance between cell self‐renewal and differentiation.^[^ ^23^ ^]^ In cancer tissues, SOX2 is often overexpressed, leading to enhanced stemness, uncontrolled proliferation, and survival of cancer stem cells. This elevated expression drives tumor growth and metastasis, contributing to more aggressive cancer behavior and is thus correlated with poor patient outcomes and prognosis.^[^ ^24^ ^]^ Moreover, SOX2 has a highly negative dependency score according to the Cancer Dependency Map (DepMap) data, which implies that cancer cell lines are highly dependent on that gene acting as an essential gene.^[^ ^25^ ^]^ In HNSCC, SOX2 drives cancer stem cells, promoting proliferation and resistance to apoptosis.^[^ ^23^, ^24^ ^]^ Therefore we hypothesize that knocking out SOX2 specifically within the tumor cells offers a potential treatment for tumor regression with minimal off‐target effects.

Herein, we report the generation of functional targeted LNPs (tLNPs), which co‐encapsulate Cas9 mRNA with sgRNA targeting SOX2 and are coated with anti‐EGFR antibodies. We assess their uptake and expression in HNSCC cancer cells as well as their therapeutic effects both in vitro and in a xenograft mouse model. This study validates SOX2 as a promising therapeutic target for HNSCC and confirms the efficacy of 𝜶EGFR‐SOX2‐CRISPR‐LNPs (cLNP) as an intratumoral therapy for visible and palpable HNSCC.

## 2 Results

### 2.1 Screening for Optimal Guide RNA for Efficient Gene Editing

**Figure 1:**
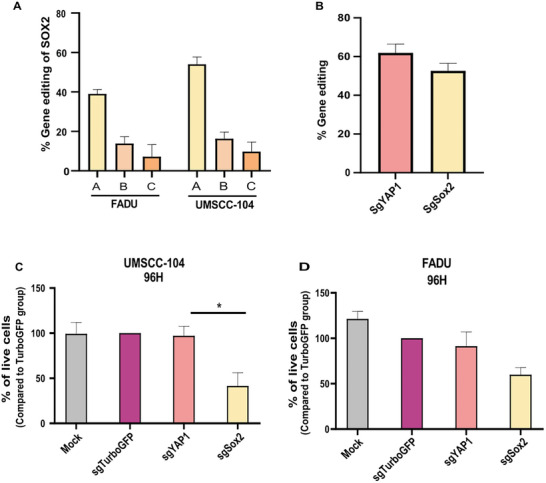
sgRNA screening and viability assays. A) Percentage of gene editing events upon RNP complex transfection with three guides for SOX2 in FADU and UMSCC‐104 HNSCC cell lines. B) Percentage of gene editing of SOX2 and YAP1 (positive control for editing) in UMSCC‐104. Data in A, and B are a representation of three independent experiments. C,D) XTT viability assay of UMSCC‐104 cells and Fadu treated with Mock, sgTurboGFP, sgYAP1, or sgSOX2 for 96 h. Bar chart representing % of cell viability normalized to sgTurboGFP‐treated cells. Data are means ±SD of three independent experiments. An unpaired T‐test was used to assess the significance. *p < 0.05.

Three sgRNAs for SOX2 (sgSOX2‐A, ‐B, and ‐C) were screened in HNSCC cell lines with different HNSCC primary origins (UMSCC‐104, FADU; **Figure** **1**A) by comparing gene‐editing efficiency after transfection with Cas9 protein and sgRNAs for SOX2 (Ribonucleoprotein/ RNP complex). The percentage of editing (Indel scores) for each designed guide was assessed by Sanger sequencing and ICE analysis. sgSOX2 (A) achieved 58% in vitro gene editing in UMSCC‐104 and 40% in FADU (Figure 1A). Hence, sgSOX2 (A) was chosen as the optimal guide for further in vitro and in vivo experiments.

To assess the effect of gene editing on cell viability, we performed XTT proliferation assays on UMSCC104 and Fadu HNSCC cells transfected with sgSOX2‐RNP. Turbo‐GFP and YAP1 sgRNAs were used as controls. YAP1, a transcription factor that induces cancer stemness and is overexpressed in HNSCC, like SOX2,^[^ ^26^ ^]^ served as a control for efficient and high editing since it caused >50% editing in two different cell lines (UMSCC‐104, FADU; Figure S1, Supporting Information). Turbo‐GFP and YAP1 sgRNAs were used to confirm that gene disruption did not affect the viability of the cells. Following cells collection 48 h post‐transfection for DNA extraction and sequencing, SOX2‐RNP resulted in 52% gene editing and YAP1‐RNP achieved 61% gene editing (Figure 1B). The viability of the remaining wells was analyzed using a plate reader at 96 h post‐transfection. The results showed a 2.5 fold reduction in UMSCC‐104 viability and a 1.6 fold decrease in FADU viability, in sgSOX2‐RNP transfected cells, compared to sgTurboGFP‐treated cultures. The preserved viability of RNP complex‐sgYAP1 treatment suggests that it does not affect cell viability despite the higher gene disruption rate (Figure 1C,D).

We also assessed the viability of UMSCC‐104 cells treated with mock, sgTurboGFP, sgYAP1, and sgSox2 ‐RNP 72 h post‐transfection by 4′,6‐diamidino‐2‐phenylindole (DAPI)/annexin V assay. A 3.2‐fold decrease in live cells compared to sgTurboGFP or sgYAP1–RNP–treated cells was reached. (Figure S2, Supporting Information) These findings underscore the effectiveness of SOX2 knockout in inhibiting tumor growth in vitro.

### 2.2 Synthesis and Characterization of CRISPR LNP Synthesis for Efficient Delivery to HNSCC Cells

**Figure 2:**
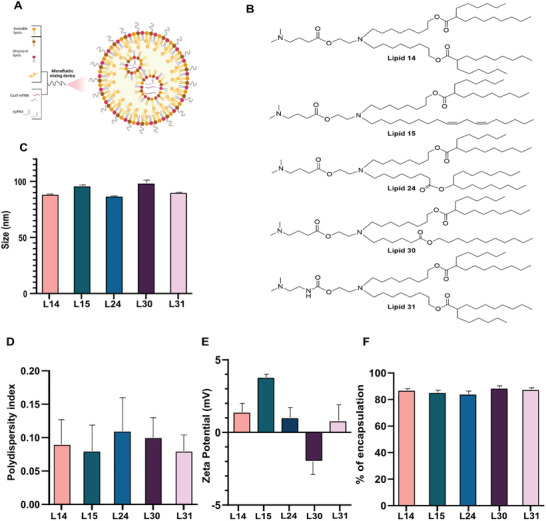
The screen of ionizable cationic lipids for the transfection of human HNSCC cells. A) Schematic illustration of LNP preparation. B) Chemical structures of selected ionizable cationic lipids from the lipid library. C) LNPs mean diameter (nm). D) polydispersity index (PDI). E) ζ potential (mV), as measured by Zeta Sizer. F) Percentage of encapsulation efficiency as measured by a RiboGreen assay. Data are means ±SD of three independent experiments.

Following the choosing of the best sgRNA for SOX2 and demonstrating its effect, we co‐encapsulated Cas9 mRNA and sgRNA in a single LNP (cLNP) for transient Cas9 protein expression (**Figure** **2**A), as we previously reported.^[^ ^8^ ^]^ To improve RNA stability and reduce immunogenicity, we employed chemically modified Cas9 mRNA with 5‐methoxyuridine and highly modified sgRNAs. We screened a library of proprietary ionizable amino lipids to determine which is the most efficient for the transfection of UMSCC‐104 cells with the combined mRNA and sgRNA. Out of the screen, lipids 14,15 were chosen as they were shown to encapsulate RNA efficiently in several of our previously published works.^[^ ^27^, ^28^ ^]^ Lipids 24, 30, and 31 are newly designed ionizable lipids, using different hydrophobic tails (See supporting information).  The structure of all used lipids 14,15,24,30, and 31 is shown in Figure 2B. LNPs were prepared using fluidic mixing at the same lipid ratio that was previously described (ionizable lipid, DSPC, cholesterol, and DSPE‐PEG at 50:10:38:0.1 molar ratio)^[^ ^8^ ^]^ and found to be uniform in size with a diameter of 80–100 nm (Figure 2C), polydispersity index of 0.02–0.17 (Figure 2D), and *ζ* potential ranging between (‐2.5) to (+4) mV as measured by dynamic light scattering (Figure 2E). The encapsulation efficiency of the sgRNA and Cas9 mRNA was similarly high in all formulations (>85%) (Figure 2F).

### 2.3 CRISPR LNPs Induce Knockout of SOX2 Leading to a Reduction in Viability In Vitro

Before evaluating the therapeutic effect of the synthesized sgSOX2 cLNPs, we wanted to assess the viability of the cells treated with cLNPs and exclude any toxicity issues. Therefore, we treated UMSCC‐104 with sgYAP1‐cLNPs and evaluated cell viability using DAPI‐Annexin assay (Figure S3A, Supporting Information). We observed that treatment with up to 10µg mL^−1^ of sgYAP1‐cLNPs did not significantly affect cell viability 72 hpost‐transfection compared to untreated cells (Figure S3B, Supporting Information). Additionally, we noted that sgYAP1‐cLNPs exhibited high indel scores for all tested cases, except lipid L24‐based cLNPs which performed with lower editing scores (Figure S3C, Supporting Information).

**Figure 3:**
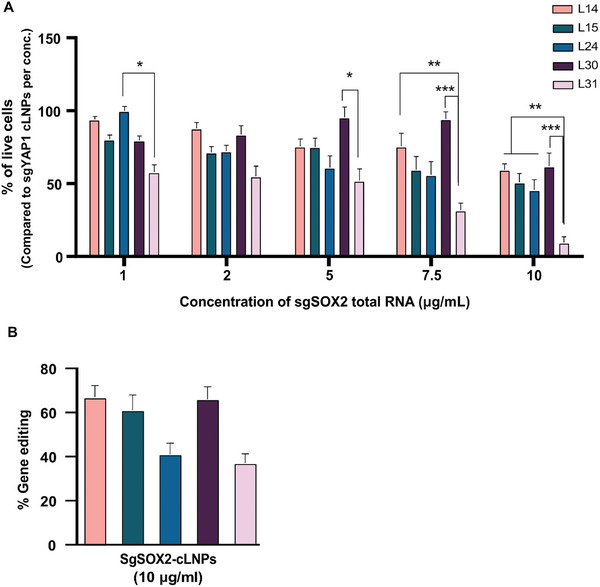
Efficacy of CRISPR‐ LNPs. A) The percentage of UMSCC cell viability 72 h post‐transfection with different concentrations of sgSOX2‐cLNP (1–10 µg mL^−1^ of total RNA) as measured by DAPI‐Annexin assay, the viability percentages of each cell treated with sgSOX2‐cLNP were normalized to those treated with the same concentration (1 to 10 µg mL^−1^ of total RNA) of sgYAP‐cLNPs. Data are means ±SD of three independent experiments. One‐way analysis of variance (ANOVA) with Tukey multiple comparison test was used to assess the significance. *p < 0.05, **p < 0.01, ***p < 0.001 B) SOX2 gene editing percentage of each formulation with the highest concentration. Data are means ±SD of three independent experiments.

Next, to evaluate the therapeutic effect of the differently formulated cLNPs (L14, L15, L24, L30, and L31). We transfected the UMSCC‐104 cells with increasing doses of sgSOX2‐cLNPs ranging from 1–10µg mL^−1^. 72 h post‐treatment, the cell viability profile was evaluated by DAPI‐Annexin assay and analyzed by flow cytometry (Figure S4, Supporting Information). The viability of sgSOX2‐cLNP at each concentration was compared to sgYAP‐cLNPs, which showed minimal impact on viability. A dose‐dependent reduction in UMSCC‐104 cell viability was observed in all formulations and achieved a maximal level of 88% cell viability reduction with L31‐based cLNP (10 µg mL^−1^), whereas L14, L15, L24, and L30‐based cLNPs (10 µg mL^−1^) showed ≈50% reduction only, relative to the effect of sgYAP1‐cLNPs (**Figure** **3**A). L31‐based cLNPs also exhibited the maximal SOX2 gene disruption with a 68% indel score by Sanger sequencing, 48 h post‐transfection (Figure 3B). Consequently, we proceeded with the ionizable lipid L31 for generating targeted cLNPs for in vivo experiments due to its superior therapeutic response, exhibiting the highest percentage of SOX2 editing and a significant reduction in UMSCC‐104 cell viability.

### 2.4 Generation of Targeted 𝜶EGFR‐sgSOX2‐cLNPs

Following the screening of various ionizable cationic lipids and selecting L31‐cLNPs, targeted LNPs were generated. Incorporation of a targeting moiety to LNPs can significantly enhance delivery efficiency and specificity to HNSCC tumor cells. Therefore, EGFR was selected as our target moiety, considering its overexpression in up to 90% of head and neck cancers.

**Figure 4:**
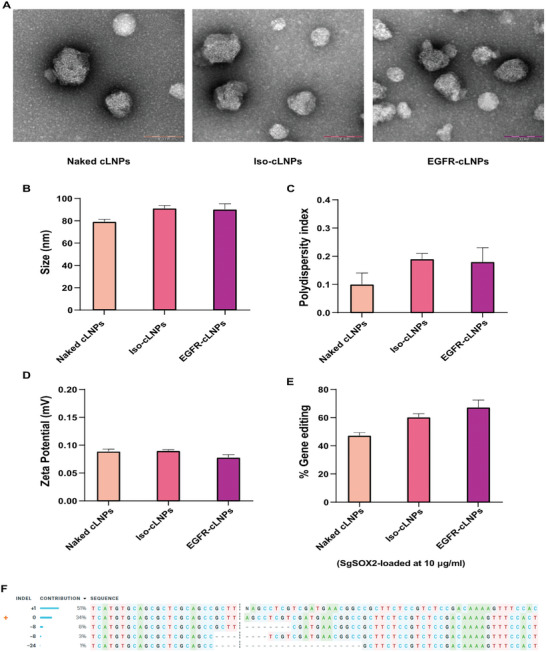
Targeted cLNP characterization and genome editing efficiency. A) Representative transmission electron microscopy of naked and tLNPs. The experiment was repeated three times independently (Scale bar = 100 µm). B) LNPs mean diameter (nm). C) polydispersity index (PDI). D) ζ potential (mV), as measured by Zeta Sizer. E) SOX2 gene editing percentage of each cLNP formulation with 10 µg mL^−1^ of total RNA, 72 h post‐transfection. Data in B‐E are means ±SD of three independent experiments. F) Indel contribution in the edited population and their edited proportions. The cut site is presented by a vertical dotted line, WT signal marked by orange +.

To generate targeted LNPs (t‐LNPs), L31‐LNPs were conjugated to an anti‐EGFR (*α*EGFR) antibody using anchored secondary scFv enabling targeting (ASSET) linker strategy for mAbs conjugation. ASSET linker is based on a recombinant protein linker that enables uniform antibody attachment in a non‐covalent method.^[^ ^8^, ^14^, ^15^ ^]^ ASSET‐LNP conjugates were coated with either anti‐human EGFR mAbs (tLNPs) or isotype antibodies (iso‐LNPs) which serve as a control for the non‐specific binding of the LNPs. An ASSET: antibody ratio of ≈1:1 forms highly stable constructs with close to 100% bio‐conjugation as previously reported.^[^ ^19^ ^]^ After tLNPs preparation, the size, uniformity, and *ζ* potential of the LNPs were evaluated. ASSET‐ antibody conjugate slightly increased the LNP mean diameter and size distribution as observed in **Figure** **4**B‐D. The uniformity of the tLNPs before and after conjugation was also confirmed by transmission electron microscopy (Figure 4A).

To confirm tLNPs genome editing efficiency, *α*EGFR‐ or iso‐tLNPs encapsulating sgSOX2 composed of lipid‐31 were used to transfect UMSCC cells in vitro. While the physiochemical characteristics of targeted and naked LNPs did not differ much, the αEGFR‐tLNPs‐sgSOX2 and iso‐tLNPs‐sgSOX2 ‐treated cells demonstrated a higher percentage of indels compared to naked LNPs (Figure 4E). Sanger sequencing and Indel analysis, (Figure S5, Supporting Information) validated the successful knockout of SOX2 with repair by NHEJ resulting mostly in insertion after the double‐strand break (DSB) (Figure 4F).

### 2.5 Uptake of Targeted LNPs by Tumor Cell In Vivo

**Figure 5:**
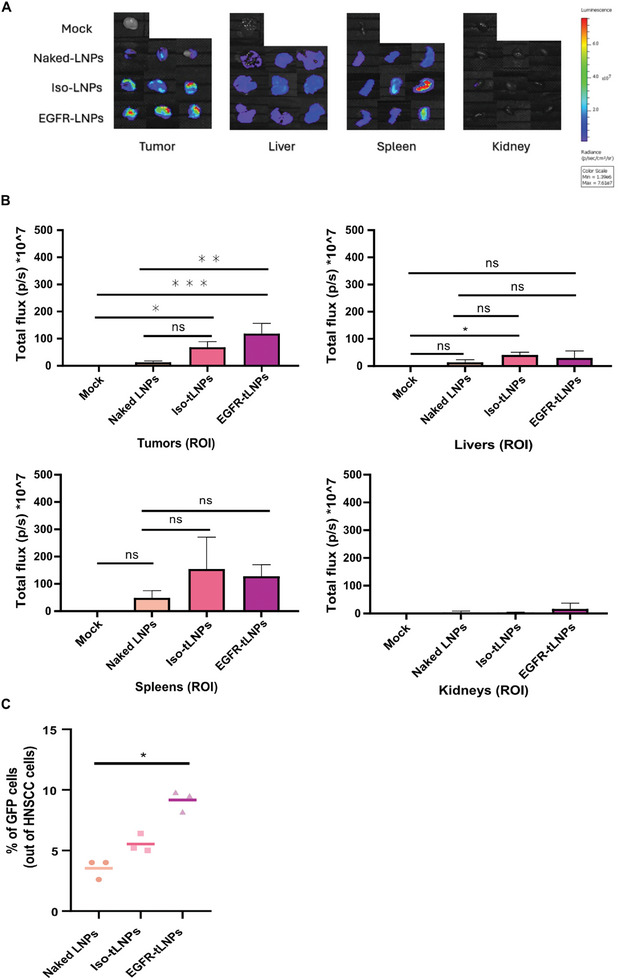
Uptake of targeted LNPs by tumor cell in vivo. A) Expression of luciferase at the tumor site, liver, spleen, and kidney at 6 h post IV injections of L31 α‐EGFR‐targeted LNPs (t‐LNPs), Isotype LNPs (Iso‐LNPs), and naked LNPs (LNPs) (n = 3 mice/group). B) Bars represent each formulation relative to the un‐injected HNSCC‐bearing mouse. Data are presented as mean ±SD; one‐way ANOVA with Tukey multiple comparison test was used to assess the significance. *p < 0.05, **p < 0.01, ***p < 0.001 C) Percentages of GFP‐positive HNSCC cells in the tumor bed, 24 h after injection of naked, isoLNPs, and α‐EGFR‐LNPs as analyzed by flow cytometry, n = 3/group. One‐way ANOVA with Tukey multiple comparison test was used to assess the significance. *p < 0.05.

A xenograft HNSCC mouse model was established and the biodistribution of intratumoral (IT)‐injected EGFR‐targeted and non‐targeted LNPs at both organ and cellular levels was evaluated. UMSCC‐104 cells were inoculated subcutaneously in 8‐week‐old female FoxN1 nude mice flanks. 10 d post‐tumor inoculation, mice were IT injected with αEGFR‐tLNPs, iso‐tLNPs, and naked LNPs encapsulating firefly luciferase mRNA (1 mg mRNA kg^−1^), 6 h later mice were sacrificed, and their organs and tumors were extracted. Bioluminescence analysis showed that αEGFR‐tLNPs achieved the highest luciferase expression in tumors with minimal leakage to spleens and livers, underscoring the importance of the anti‐EGFR antibody for targeting (**Figure** **5**A,B).

To determine the intra‐tumoral distribution of the Luciferase‐LNPs, cryosections were performed and stained for anti‐luciferase and anti‐EGFR (tumor cells marker) (Figure S6A, Supporting Information). Both isoLNPs and tLNPs were widely expressed throughout the tumors, indicating comparably successful extravasation through tumor vessels. However, High‐power magnification revealed distinctive patterns of intra‐tumoral distribution for αEGFR‐tLNPs compared to isoLNPs. We observed stronger anti‐luciferase stains in the tumor cells (EGFR stained) with the tLNPs compared to the isoLNPs cancer samples (Figure S6B, Supporting Information).

A cellular‐level biodistribution with tLNPs, isoLNPs, and naked LNPs encapsulating GFP‐mRNA following IT injection was evaluated to analyze the mRNA expression quantitatively. Tumors were extracted 24 h post‐injection and analyzed by flow cytometry for expression of GFP in UMSCC‐104 CD44+ cells (Figure S7, Supporting Information). Significantly higher levels of GFP were detected in human CD44+ cancer cells injected with 𝛼EGFR‐tLNPs compared to naked or iso‐tLNPs. (**Figure** **5**C). These results highlight the efficiency of anti‐EGFR antibody targeting in reaching the tumor cells and the importance of intratumoral injection to minimize the off‐target effects.

**Figure 6:**
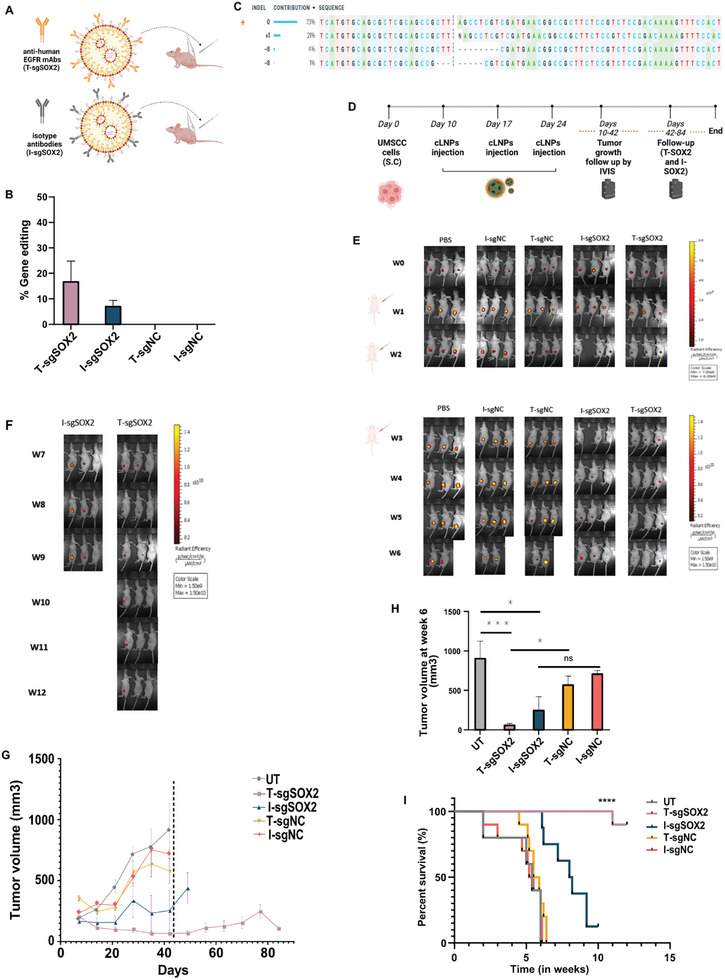
αEGFR‐SOX2‐LNP mediates therapeutic gene editing in a xenograft HNSCC mouse model. A) Schematic illustration of targeted cLNPs preparation and IT injection with Debakey tweezers and Kel‐F‐needle inserted to Hamilton syringe. B) SOX2 gene editing percentage events 5 days after injection of Iso or 𝜶EGFR‐T‐sgSOX2‐cLNPs or sgNC (scramble control guide), (n = 3 mice/group). C) Indel's contribution to the edited population and their edited proportions. D) Experimental design. UMSCC‐104 cells were subcutaneously inoculated in FoxN1 nude mice. After tumors had reached ≈50 mm^3^ (day 10), mice were IT injected with 𝜶EGFR‐LNPs or iso‐LNPs encapsulating sgSOX2 or sgNT on days 10,17,24, n = 10 mice/group and mock group injected with BPS (n = 5). E and F) Representative fluorescence imaging of UMSCC‐bearing mice for 6 weeks for all groups and long‐follow of 12 weeks for 𝜶EGFR‐LNPs encapsulating sgSOX2. G) Tumor growth inhibition by triple‐dose treatment with cLNPs, data are presented as means ± SEM; H) Tumor volume of each treatment group at week 6, data presented as means ±SD; one‐way ANOVA with Tukey multiple comparison test was used to assess the significance. *p < 0.05, **p < 0.01, ***p < 0.001. I) Survival curves of UMSCC‐bearing mice. n = 10 animals per treatment group, showing 3 representative mice per cage. ****p < 0.0001. Log‐rank (Mantel‐Cox) test was used for curve comparison.

Next, we aimed to evaluate whether the specific targeting of tumors by *α*EGFR‐cLNPs can cause gene editing in the tumor cells in vivo. To this end, mice bearing UMSCC‐mCherry tumors were IT injected, 10d post‐tumor inoculation, with either I/ or T‐sgNC (scramble control guide (sgNC)), or sgSOX2‐cLNPs (1 mg kg^−1^) (**Figure** **6**A). Mice were sacrificed 5d later; tumors were collected and dissociated to single‐cell tumor suspensions. Then, the tumor cells were analyzed for gene editing percentages in the SOX2 knockout. Results showed that a single treatment with T‐sgSOX2‐cLNPs facilitated ≈17% gene editing in the SOX2 locus, (Figure 6B,C) (Figure S8, Supporting Information), while less than 5% gene editing was detected in T‐sgNC‐cLNP and no editing in all other treatment groups (Figure 6B).

Considering that a small fraction of our LNPs might reach the liver and spleen following intratumoral injection as seen in our biodistribution results, spleen and liver cells were analyzed for gene editing percentages in the SOX2 knockout, which confirmed no off‐target editing due to the IT injection (Figure S9, Supporting Information).

Furthermore, we evaluated liver toxicity, and serum levels of inflammatory cytokines 24 and 48 h after intravenous injection of sgSOX2‐cLNPs (1 mg kg^−1^) into nude mice. There were no apparent clinical signs of toxicity and no significant difference in liver enzyme levels (alanine transaminase, aspartate aminotransferase, and alkaline phosphatase) levels (Figure S10A, Supporting Information) or kidney function parameters (Figure S10B, Supporting Information). In addition, a plasma cytokine panel [TNFα, IL‐10, and IL‐1β] also showed no significant differences (Figure S10C, Supporting Information). Overall, these results suggest that SOX2‐cLNPs are not toxic or immunogenic.

### 2.6 Efficacy of 𝜶EGFR‐SOX2‐LNPs in HNSCC‐Bearing Mice

To explore the ability of our *α*EGFR‐tLNPs platform to mediate therapeutic gene editing in vivo, we evaluated the inhibition of tumor growth and survival of the mice. For this end, mice bearing UMSCC‐mCherry tumors were IT injected when the tumors had reached a volume of ≈50 mm^3^, at days 10, 17, and 23 post‐tumor inoculation, with 1 mg kg^−1^ of either sgSOX2‐*α*EGFR‐tLNPs (T‐sgSOX2), sgSOX2‐iso‐LNPs (I‐SOX2), sgNC‐*α*EGFR‐tLNPs (T‐sgNC), or sgNC‐*iso‐*LNPs (I‐sgNC) (Figure 6D, 10 mice/group). Five mice were injected with phosphate buffer saline (PBS) in a similar treatment regimen. Tumor growth was monitored using mCherry live animal fluorescent imaging up to 42d post‐tumor inoculation. Mice with tumors reaching 1500 mm were sacrificed. (Figures 6E,F; Figure S11, Supporting Information).

The results showed that triple treatment with T‐sgSOX2‐cLNPs inhibited tumor growth by 90% compared to I‐sgNC (Figure 6G,H; Figure S12, Supporting Information). Additionally, no significant difference in tumor growth or survival was observed in control mice treated with either T‐sgNC, I‐sgNC, or PBS. (Figure 6H,I) We continued the long‐term tumor growth follow‐up to 84d with sgSOX2 groups (T‐SOX2 and I‐SOX2) and observed a regrowth in only one of the 10 mice of the sgSOX2 groups, which reached a tumor of 1500 mm and was therefore sacrificed. T‐SOX2 tumor growth inhibition increased the overall survival of the mice by ≈90% (from 42 to >84 days) (Figure 6I). Tumor growth was also strongly inhibited by I‐sgSOX2‐treated mice for 6 weeks, but tumors tended to regrow (>6 weeks), unlike T‐SOX, which showed a sustained response (Figure 6F (representative fluorescence imaging) and Figure S11 (Supporting Information) (original fluorescent imaging)), highlighting the sustainable therapeutic effect of the T‐sgSOX2‐cLNPs.

Overall, these findings suggest that intratumoral administration of targeted cLNPs may be an effective therapeutic approach for accessible solid tumors.

## 3 Discussion

Over the past few years, mRNA‐Cas9‐LNPs, known for their efficient loading and flexible design have seen widespread use in clinical‐stage CRISPR therapies for various diseases. At the same time, oncology has become the leading indication for newly FDA‐approved therapies,^[^ ^29^ ^]^ prompting preclinical research into utilizing mRNA‐Cas9‐LNPs for solid cancer therapy.^[^ ^8^, ^9^, ^30^, ^31^, ^32^ ^]^ Yet, LNPs' liver accumulation, rapid blood clearance, and efficient cancer cell specific‐targeting can pose challenges affecting gene editing efficiency.^[^ ^10^, ^21^ ^]^ Therefore, incorporating additional layers of organ and cellular specificity into the CRISPR‐Cas9 delivery system is essential for effectively targeting cancer cells while minimizing off‐target effects.

In this study, we developed an effective method for IT injection of EGFR‐targeted CRISPR LNPs (cLNPs) that knock out a cancer‐specific gene, SOX2. Selecting a tumor‐specific target gene, employing IT administration to enhance intratumoral accumulation and retention, and decorating LNPs with target moieties for active targeting and internalization into tumor cells, collectively enhance the specificity and safety of CRISPR technology in cancer applications. This methodology is particularly applicable for visible, palpable, and accessible solid tumors like HNSCC.

LNPs' distribution within solid tumor masses depends on their extravasation from tumor vasculature.^[^ ^7^, ^33^ ^]^ Studies suggest that the presence of targeting moieties does not significantly affect this process, as both targeted and nontargeted LNPs can passively extravasate through the leaky vasculature of solid tumors and benefit equally from the enhanced permeability and retention (EPR) effect.^[^ ^7^, ^34^ ^]^ However, active targeting is hypothesized to enhance LNPs' retention at the intended site by facilitating stronger binding to tumor cells, leading to increased accumulation and cellular uptake through receptor‐mediated endocytosis.^[^ ^35^, ^36^ ^]^ Our analysis of intratumoral biodistribution in mice with tumors, following IT injection of luciferase or GFP mRNA encapsulated in targeted and nontargeted LNPs, supported the hypothesis. EGFR‐targeted LNP resulted in higher expression of luciferase in the tumor bed, along with a prominent immunofluorescent‐staining signal when stained for luciferase antibody, compared to naked LNPs or isotype‐LNPs. Similar results were observed with IT injection of EGFR‐targeted LNPs encapsulating GFP mRNA, showing higher GFP expression in CD44‐tumor cells compared to naked LNPs or isotype‐LNPs.

Regarding the efficacy of EGFR‐targeted cLNPs, a significantly inhibited tumor growth by 90% was observed in T‐sgSOX2‐cLNPs (T‐sgSOX2) compared to I‐sgNC‐cLNPs (I‐sgSOX2) treated mice. This tumor growth inhibition was sustained for >12 weeks. T‐sgNC‐cLNPs demonstrated a certain degree of tumor growth inhibition compared to the I‐sgNC‐cLNPs group. This therapeutic effect is attributed to EGFR antibodies, indicating that these antibodies are not inert and may actively enhance the therapeutic effect of the delivered cargo.

Ectopic expression of a constitutively active EGFR mutant or ligand exposure, same as HNSCC cancer, enhances STAT3 nuclear translocation and binding to the SOX2 promoter, leading to increased SOX2 expression to facilitate cell survival and self‐renewal of cancer cells.^[^ ^23^, ^37^ ^]^ Inhibition of EGFR signalling, through pharmacological inhibition or genetic inactivation, significantly reduces the SOX2 expression and subsequently suppresses the self‐renewal of cancer stem‐like cells.^[^ ^23^ ^]^ Our efficacy results demonstrated a synergistic effect when combining SOX2 knockout and EGFR targeting, with significantly higher inhibition of tumor growth and persistent response achieved by the combined approach (T‐sgSOX2) compared to each component separately (I‐sgSOX2 or T‐sgNC‐LNPs).

The improvement in the delivery of LNP due to utilizing the EGFR antibodies as targeting moieties resulted in an increased level of gene editing in tumor cells mediated by CRISPR‐Cas9. This increase in gene editing not only enhanced the therapeutic effectiveness but also significantly prolonged mouse survival, with all ten (100%) mice surviving beyond 42 days, compared to only 2 mice (40%) in the PBS‐treated group and 4 mice (40%) of I‐sgNC‐LNPs surviving until the end of the six weeks. About half of patients with locally advanced disease will develop recurrence, a primary cause of treatment failure in HNSCC. Therefore, we conducted imaging and survival follow‐ups until the end of week 12. The most significant outcome was the sustained therapeutic response in the T‐SOX2 group, with 3 mice showing complete response (tumor bed disappearance) at week 6 and 5 mice at week 12.

One mouse in the T‐SOX2 group experienced tumor recurrence, which may be attributed to several factors. First, epigenetic changes could activate other stem cell‐related genes, enabling the tumor cells to compensate for the SOX2 inhibition. Second, due to the heterogenous nature of tumors different animals may have tumor subpopulations that do not rely on SOX2 and thus may survive treatment, leading to resistance. Finally, components in the tumor microenvironment, such as cancer‐associated fibroblasts and immune cells, could foster resistance by supporting cell survival and restoring stem cell‐like properties independent of SOX2. Identifying biomarkers that predict resistance could also aid in personalizing treatments and selecting patients most likely to benefit from SOX2 inhibition.

Using intratumoral cLNP injections allowed for highly concentrated delivery at the tumor site while minimizing systemic exposure. Several important considerations are essential for achieving a successful treatment response. First, it is essential to begin injections before the tumor solidifies. We followed established guidelines for intratumoral drug administration in solid tumor clinical trials, which recommend caution with very large tumors due to the risks of central necrosis, increased bleeding, and challenges in effective drug distribution. Failing to administer treatment within the optimal window—when the tumor volume in our model is typically between 50–100 mm^3^—could lead to treatment failure. Second, for recurrent intratumoral administration of LNPs to the solid tumor bed, using a fine needle (Kel‐F‐31 gauge) is vital to avoid puncturing the tumor. The established guidelines prioritize superficial and visible lesions, making them well‐suited for our UMSCC‐104 oral cavity HNC, which is easily accessible with a needle. In a clinical setting, deeper lesions can be targeted using specialized needles, often under endoscopic guidance with imaging support such as ultrasound. Thus, this limitation of deep lesions is more related to equipment and the expertise of the professional administering the treatment rather than any inherent issues with the treatment itself. Regarding the LNP dose, systemic administration typically considers mouse weight, while intratumoral LNPs can be dosed based on tumor volume or weight. In our experiment, we dosed based on mouse weight (0.025 mg of LNPs per mouse) necessitating LNP concentration in low volumes.  Nonetheless, it's important to note that the optimal dose and schedule for intratumoral LNPs may vary depending on the safety profile of the therapeutic RNA payload, the targeting moiety, or the combination of agents encapsulated within the LNPs. Further experiments with more intense treatment protocols may refine our findings.

Our highly specific intratumoral T‐SOX2‐cLNPs offer advantages in both effectiveness and safety, however, this approach is particularly suitable for targeting easily accessible tumors such as those in HNSCC, breast, thyroid, and skin cancer, or cancers with a cancer‐specific target gene, exclusively expressed in cancer cells, crucial for their survival, and associated with poor prognosis and adverse clinicopathological characteristics, like SOX2. Hence, this study proposes a simple approach that brings DSB platforms closer to clinical applications, particularly for clinically accessible solid tumors like HNSCC. However, DSBs may occur in partially similar sequences in the genome to the desired CRISPR target, leading to undesired genetic alterations, known as off‐targets,^[^ ^10^, ^22^ ^]^ which pose significant risks to the safe clinical application of CRISPR LNPs. These concerns have driven efforts to develop DSB‐free platforms, such as base editors (BEs) and prime editors (PEs), aimed at enhancing CRISPR safety. While offering advantages over DSB‐gene‐editing technologies, base editors (BEs) are limited to introducing specific point mutations and may still result in some unwanted modifications, potentially leading to off‐target effects.^[^ ^38^, ^39^ ^]^ In contrast, prime editors (PEs) can create precise insertions and deletions with minimal unwanted modifications and virtually no off‐target effects.^[^ ^40^, ^41^ ^]^ Therefore, incorporating targeted delivery with prime editors may hold promising potential for the precise targeting and correction of a wide range of oncogenic mutations.^[^ ^42^ ^]^ Thus, there are still numerous challenges to overcome for the widespread application of CRISPR techniques across diverse types of solid cancers.

Finally, in clinical practice, unresectable advanced HNSCC patients are seldom treated with a solitary therapeutic approach, with most regimens incorporating a combination of radiotherapy, immunotherapy, or chemotherapy.^[^ ^3^ ^]^ While our T‐sgSOX2‐cLNPs demonstrated a significant therapeutic impact; enhancing it further may necessitate combination strategies with different therapeutic RNA molecules (siRNA against SOX2 oncogene or other prognosis‐related oncogenes such as HPV oncogenes) within the same tLNPs. The use of these multiplexed LNPs could yield a potent effect even with reduced doses.

## 4 Conclusion

We reported pre‐clinical evidence for successful and prolonged tumor growth inhibition in HNC achieved by Cas9 mRNA and guide against SOX2 administered intratumorally via EGFR‐targeting LNPs. Ultimately, this highly specific treatment strategy that includes cancer‐specific target gene SOX2 with functional anti‐EGFR tLNPs holds great promise for safe and efficient CRISPR treatment HNC, and ultimately for many other palpable solid cancers. Specifically, in this model of solid HNC, the improved therapeutic effect of the tLNPs compared to isoLNPs can be explained by several mechanisms: higher LNP retention and greater increased accumulation, or the synergism of knocking out SOX2 and inhibiting EGFR activation and its downstream signaling which subsequently suppresses the self‐renewal of cancer stem‐like cells modulation of SOX2‐oncogene.

## 5 Experimental Section

### Cell Culture

FaDu hypopharyngeal carcinoma cell line (ATCC, USA) and the UMSCC‐104 oral cavity carcinoma cell line (Millipore, USA) were maintained in DMEM (Gibco, Thermo‐Fisher Scientific, Inc) supplemented with 10% FBS (Biological Industries, Israel), 1% L‐glutamine (Gibco, Thermo‐ Fisher Scientific, Inc), and 1% Penicillin‐Streptomycin‐Nystatin (Biological Industries, Israel). Cells were grown and maintained in a humidified incubator at 37 °C with 5% CO_2_ (Thermo, MA, USA). All cells were routinely checked every month for mycoplasma contamination using the EZ‐PCR Mycoplasma Test Kit (Biological Industries, Israel) or Hy‐Mycoplasma Kit (HyLabs, Israel) according to the manufacturer's protocols.

### RNP Complex Transfection

To screen for an optimal single‐guide RNA, which will lead to efficient SOX2 gene editing, each guide (sgRNA XT, IDT) was mixed with cas9 protein (IDT), this Cas9‐gRNA complex, known as ribonucleoprotein (RNP) complex, was transfected with FADU, and UMSCC‐104, according to IDT protocol and collected after 48 post‐transfection and prepared for sequencing.

### RNA Sequences

sgRNAs were designed and synthesized by Integrated DNA Technologies (IDT)

HsSOX2 guide A: CGTTCATCGACGAGGCTAAG

HsSOX2 guide B: GATAAGTACACGCTGCCCGG

HsSOX2 guide C: CTCACGTCGTAGCGGTGCAT

HsYAP1 guide: TCGAACATGCTGTGGAGTCA

CleanCap Cas9 mRNA (modified) was purchased from TriLink BioTechnologies Inc.

### Sanger Analysis of Gene Editing

For sequencing analysis to determine the gene‐editing percentage, genomic DNA was first extracted with QuickExtract DNA Extraction Solution (Lucigen Inc) according to the manufacturer's protocol. Then, amplification was performed using SOX2‐locus‐specific primers (HsSOX2 primers, forward: GATGGAGACGGAGCTGAAGCC, Reverse: TCAAGTCCGAGGCCAGCTC, or Reverse: GGATAAGTACACGCTGCCCGG) in a round of PCR. After gel electrophoresis, amplified DNA was purified with the Monarch DNA Gel Extraction Kit (Biolab monarch PCR & DNA clean‐up) before sequencing. Sanger sequencing services were provided by HyLabs. Acquired files were uploaded to the ICE web tool (Synthego) and analyzed for Indel formation.

### Cell Proliferation by XTT Assay

Cell proliferation was evaluated using the XTT cell proliferation kit (Biological Industries, Israel) according to the manufacturer's protocol. Briefly, 15^4^ cells/100 µl were seeded in a 96‐well plate and treated with either Medium or 1–10µg mL^−1^ of sgYAP or sgSOX2‐RNP complex. 72–96 h post‐ RNP transfection, 50 µl of reaction solution containing activation solution, and XTT reagent (1:50 ratio by protocol) were added to each well. The plate was incubated at 37 °C for 2 h, and the absorbance of the samples against a background control was measured by a plate reader (Biotek Industries) at a wavelength of 450–500 nanometers subtracting the reference absorbance (at a wavelength of 630–690 nanometers).

### Preparation of LNPs

Ionizable lipids (Lipids 14,15) were synthesized and designed as previously described.^[^ ^27^, ^28^ ^]^ Lipids 24, 30, and 31 were newly designed ionizable lipids, using different hydrophobic tails (see supporting information).

The structure of all used lipids 14,15,24,30, and 31 is shown in Figure 2B. Cholesterol, DSPC, and PEG‐DMG were purchased from Avanti Polar Lipids Inc. Briefly, one volume of lipid mixture (Ionizable lipid: DSPC: Chol: DMG‐PEG at   50:10:38:2 mol ratio) in ethanol was mixed with three volumes of mCas9 (1 mg mL^−1^)/sgRNA (2 mg mL^−1^) (1:10 molar ratio RNA to ionizable lipid) in a citrate buffer (pH = 5) by the NanoAssemblr (Precision  Nanosystems Inc. Canada), a microfluidic mixing device. LNPs encapsulating Luciferase were prepared likewise with similar RNA: ionizable lipid molar ratio. After LNPs generation, the formed particles were dialyzed twice in Maxi GeBAflex tubes (GeBa, Israel) against phosphate‐buffered saline (PBS) (pH 7.4) overnight to remove ethanol. LNPs were conserved in glass vials at 4 °C.

### ASSET Incorporation to LNPs and Targeted LNP Production

To incorporate ASSET into LNPs, ASSET was prepared as previously described.^[^ ^8^, ^14^, ^15^ ^]^ Then, the cLNPs were mixed with ASSET micelles (4 ug of ASSET for 100 uL of LNPs) and incubated for 48 h at 4 °C to allow its incorporation into LNPs. Next, to construct antibody‐targeted cLNPs, anti‐human EGFR antibody (MCA1784, clone ICR10, Bio‐Rad, USA) or Rat IgG2a isotype control (BioXcell NH, USA. Clone 2A3) were added to the ASSET incorporated cLNPs (1:1, mAbs: ASSET weight ratio) and incubated for 30 min at room temperature.

### LNPs Characterization: Size Distribution and ζ‐Potential Measurements

The size distribution (hydrodynamic diameter) and ζ‐potential of the cLNPs were measured by dynamic light scattering using a Malvern nano ZS ζ‐sizer (Malvern Instruments Ltd., Worcestershire, UK). For size measurements, cLNPs were diluted 1:20 in PBS in a DTS0012 sizing cuvette (Sarstedt, Germany). All cLNPs preparations showed a polydispersity index (PDI) lower than 0.2. For ζ‐potential measurements, cLNPs were diluted 1:200 in double‐distilled water (DDW) in DTS1070 zeta cuvettes (Malvern Instruments Ltd., Worcestershire, UK).

### Transmission Electron Microscopy

Transmission electron microscopy (TEM) visualization was performed by a drop of an aqueous solution containing cLNPs placed on a carbon‐coated copper grid, dried, and analyzed using a JEOL 1200 EX transmission electron microscope (Jeol, Japan).

### CRISPR LNPs (cLNPs) Quantification and Encapsulation Efficiency

To quantify cLNPs and assess RNA encapsulation efficiency, the Quant‐iT RiboGreen RNA assay (Life Technologies, CA, USA) was employed following the manufacturer's instructions. Briefly, 2 µl of cLNPs or ribosomal RNA (utilized for the standard curve) was mixed with TE buffer in a 96‐well fluorescent plate (Costar, Corning, NY, USA) to a final volume of 100 µl. The mixture was prepared both with and without 0.5% Triton X‐100 (Sigma–Aldrich). After a 10‐min incubation at 40 °C for cLNP permeabilization, a blend of 99 µl TE buffer and 1 µl RiboGreen reagent was added to each sample. Following a 5 min room temperature shake, fluorescence (excitation wavelength 485 nm, emission wavelength 528 nm) was measured with a plate reader (Biotek Industries) according to the manufacturer's guidelines.

### cLNPs Transfection

For all in vitro studies, cells were seeded in 96‐well tissue culture plates (Greiner bio‐one, Germany) in the corresponding media with 1–10µg mL^−1^ of cLNPs added to the cells and incubated for 48 h h in standard culture conditions. Then, cells were washed, trypsinized, and collected for further investigation. For DNA purification and Sanger analysis, cells were collected 48 h post‐transfection. For cell viability analysis by both XTT and DAPI/Annexin V, cells were collected 72–96 h post‐transfection.

### Cell Viability Posy cLNPs Transfection by Flow Cytometry

For cell viability evaluation, 1.5 × 10^5^ cells per well were seeded in a 12‐well plate in the corresponding growth media. Cells were treated with either DMEM media or 1–10µg mL^−1^ of sgYAP or sgSOX2‐ cLNPs. Cells were harvested 72–96 h post‐LNPs transfection and dyed with AnnexinV‐APC (Biolegend, Inc: 640941) and DAPI as recommended by the manufacturer. Data from cells were acquired and analyzed using Cytoflex and the Cytexpert software (Beckman‐Coulter, USA).

### Animal Experiments

All animal studies were performed in accordance with the protocols approved by the ethics committee (Protocol # TAU–LS–IL–2310–158–5) by the Tel Aviv University Institutional Animal Care and Usage Committee and by current regulations and standards of the Israel Ministry of Health. Mice were randomly divided in a blinded fashion at the beginning of each experiment.

### UMSCC‐104 Bearing Mice

For all head and neck cancer in vivo experiments, eight‐week‐old female Athymic Nude‐Foxn1 mice (Envigo, Rehovot, Israel) were used. The mice were housed in groups of five in cages within an SPF facility and maintained on a 12‐h day/night cycle at 23 °C. Mice were always given free access to food and water. Mice were anesthetized with ketamine/xylazine solution (200 mg ketamine and 20 mg xylazine in 17 mL of saline) at a dosage of 15 mg kg^−1^ body weight. Then, mice flanks were injected subcutaneously with 5 × 10^5^ UMSCC‐ mCherry cells in 40 µl volume of PBS and 40 µl of Matrigel. Fluorescence imaging (IVIS‐spectrum‐CT Perkin Elmer Inc) was performed weekly after tumor cell implantation to monitor tumor growth.

### Concentrated CRISPR LNPs (cLNPs) for In Vivo IT Injection

Amicon Ultra‐0.5 100K centrifugal filters (Millipore) were used to concentrate LNPs to a final volume of 50 µl.

### In vivo Tumor Bioluminescence Biodistribution

For cancer cell‐targeting and biodistribution experiments, UMSCC‐bearing mice were injected intratumorally with concentrated 1 mg Kg^−1^ of either anti‐EGFR mLUC‐LNPs, Isotype control mLUC‐LNPs, or naked LNPs. Fluorescence imaging (IVIS‐spectrum‐CT Perkin Elmer Inc) was performed 6 h after LNP injection to evaluate tumor targeting and accumulation. Fluorescence analysis was conducted using the Living Image software (Perkin Elmer Inc).

Six hours post‐injection mice were processed for immunostaining. Briefly, tumors were extracted and incubated with 10% formalin overnight at 4 °C. Then, the tumors were transferred to 15% and 30% sucrose overnight at 4 °C until sedimentation. Finally, the tumors were embedded in OCT and stored at −80 °C. Coronal brain sections in a (12µm) were cut on a microtome.

After processing the tissues were incubated overnight with anti‐LUC (GTX125849, GeneTex) and anti‐EGFR antibody (MCA1784, Bio‐Rad). Slides were then washed with PBS and incubated with Alexa‐Fluor 488 labeled secondary antibody (ThermoFisher Scientific, USA) or Alexa–Alexa‐Fluor 495 labeled secondary antibody (Jackson immunoResearch, USA) for 1 h at room temperature, and underwent DAPI immune‐mounting (Moshe Stauber Biotec Applications, Israel). Dried slides were examined and imaged by a TCS SP8 multiphoton confocal microscope (Leica, USA). Analysis of fluorescent colour mean stain intensity was conducted using the ImageJ program.

### In vivo Tumor Bio‐Fluorescence Biodistribution

UMSCC‐bearing mice were injected intratumorally with 1 mg Kg^−1^ of either anti‐EGFR GFP‐LNPs, Isotype control GFP‐LNPs, or naked LNPs. 24 h post LNP injection mice were sacrificed and single‐cell suspension was performed as described below, were collected, washed, and stained with PE‐Cy7 α‐human CD44 (Biolegend, USA) and analyzed for their GFP fluorescence signal by flow cytometry. The percentage of GFP‐positive CD44+ human UMSCC tumor cells was evaluated using Cytoflex (Beckman‐Coulter, USA). The percentage of GFP‐positive cells upon treatment (anti‐EGFR GFP‐LNPs, Isotype control GFP‐LNPs, or naked LNPs) were compared in each tumor cell and immune cells to mock (PBS) treated control.

### cLNPs Intratumoral Injections

UMSCC‐bearing mice were injected intratumorally with 1 mg Kg^−1^ of either anti‐EGFR‐sgNC‐ cLNPs, Isotype control‐sgNC‐cLNPs, anti‐EGFR sgSOX2‐cLNPs, or Isotype control‐sgSOX2‐ cLNPs. To ensure distribution within the tumor bed without leakage subcutaneously, DeBakey forceps were used to elevate and hold the tumor bed before injection as depicted in Figure 6A. A special microliter syringe (Hamilton, Model 705 LT SYR) with a fine needle (Kel‐F‐31 gauge) was used to avoid puncturing the tumor.

### Generation of Tumor Single‐Cell Suspensions and Gene Editing

For sequencing analysis, 5 days post‐injection three mice were sacrificed from each treatment or control group and tumor beds were extracted, washed with PBS, and processed to single‐cell suspensions using the Tumor Tissue Dissociation Kit, mouse (Miltenyi Biotech, USA), and gentleMACS Dissociator (Miltenyi Biotech, USA) according to the manufacturer's protocol. Briefly, tumors were extracted, and tumors were cut into small pieces of 2–4 mm and transferred to a gentle MACS C Tube containing enzyme mix. The tube was placed in the gentleMACS Dissociator for a short run, and samples were incubated for 40 min at 37 °C under slow continuous rotation for another dissociation then the cell suspension was strained using a 70 µm strainer, centrifuged and red blood cells were lysed by Red Blood Cell Lysis Solution (10×). DNA extraction and Sanger sequencing were performed as described above.

### In Vivo Toxicity and Immunogenicity

Ten‐week‐old female nude‐Foxn1 mice (Envigo Laboratories) were injected with either EGFR‐sgSOX2‐cLNPs, Iso‐sgSOX2‐cLNPs, naked sgSOX2‐cLNPs, and PBS at a dosage of 1 mg kg^−1^ intravenously (n = 8/group). 24 and 48‐ h after injection (n = 4/group at each timepoint), blood was collected for biochemistry using a Cobas‐6000 instrument and complete blood count via Sysmex and ADVIA 120. The serum was separated and stored at −80 °C before cytokine analysis.

### Tumor Growth and Efficacy Studies

10 d post tumor inoculation (≈50 mm^3^ tumor volume), UMSCC‐bearing mice were intratumorally injected with 1 mg Kg^−1^ of either anti‐EGFR‐sgNC‐ cLNPs, Isotype‐control‐sgNC‐cLNPs, anti‐EGFR sgSOX2‐cLNPs, or Isotype‐control‐sgSOX2‐ cLNPs, with 10 mice per group, using a special microliter syringe (Hamilton, Model 705 LT SYR) with a fine needle (Kel‐F‐31 gauge) to avoid puncturing the tumor.  The IT injections were repeated weekly for three weeks (3 injections in total).

Tumor growth was monitored twice a week by IVIS imaging. Fluorescence analysis was conducted using the Living Image software (Perkin Elmer Inc). Tumor volume was measured with an electronic caliper. Cephalo‐caudal length (L) and mediolateral dimensions (W) were recorded, and tumor volume (V) was calculated by the following formula: V = (L x W^2^)/2. For survival analysis, Kaplan–Meier curves were created with Prism 7 (GraphPad Software).

### Statistical Analysis

Statistical analysis for comparing two experimental groups was performed using two‐sided Student's t‐tests. In experiments with multiple groups, one‐or two‐way analysis of variance (ANOVA) with a Tukey correction was used to calculate differences among multiple populations. Kaplan–Meier curves were used to analyze survival. A value of *p* < 0.05 was considered statistically significant. Analyses were performed with Prism 8 (GraphPad Software). Differences were labeled n.s. for not significant, * for *p* ≤ 0.05, ** for *p* ≤ 0.01, *** for *p* ≤ 0.001, and **** for *p* ≤ 0.0001. Pre‐established criteria for the exclusion of animals from the experiment were based on animal health, behavior, and well‐being as required by ethical guidelines.

## Conflict of Interest

D.P. receives licensing fees (to patents on which he was an inventor) from, invested in, consults (or on scientific advisory boards or boards of directors) for, lectured (and received a fee), or conducts sponsored research at TAU for the following entities: ART Biosciences, BioNtech SE, Earli Inc., Geneditor Biologics Inc.; Kernal Biologics, Newphase Ltd., NeoVac Ltd., RiboX Therapeutics, Roche, Teva Pharmaceuticals Inc. All other authors declare no competing financial interests.

## Supporting information



Supporting Information

## Data Availability

The data that support the findings of this study are available from the corresponding author upon reasonable request.
