# Supplementary material for: Targeted CRISPR/Cas9 Lipid Nanoparticles Elicits Therapeutic Genome Editing in Head and Neck Cancer
Source: Adv Sci (Weinh). 2024 Dec 30;12(7):2411032. doi: 10.1002/advs.202411032 (PMC11831472; doi:10.1002/advs.202411032)
Supplement: Supplementary file 1 — Supporting Information [file ADVS-12-2411032-s001.docx]

Targeted CRISPR/Cas9 Nanomedicine Elicits Therapeutic Genome Editing in Head and Neck Cancer.

Razan Masarwy, Dor Breier, Lior Stotsky-Oterin, Nitay Ad-El, Shahd Qassem, Gonna Somu Naidu, Anjaiah Aitha, Assaf Ezra, Meir Goldsmith, Inbal Hazan-Halevy, Dan Peer*

Supporting Information

Supporting Information is available from the Wiley Online Library or from the author.

**Lipid synthesis**

**General Information**: All reactions were performed in oven-dried (120 °C) glass apparatus. All the chemicals were purchased from Sigma Aldrich unless mentioned. Linoleyl alcohol was obtained from TCI chemicals. Anhydrous dichloromethane (CH_2_Cl_2_) and tetrahydrofuran (THF) were purchased from Aldrich. Thin Layer Chromatography (TLC) was carried out using Merck silica gel 60 F_254_ plates. Column chromatography was performed on silica gel 60A (0.063-0.2 mm). ^1^H NMR spectra were recorded on a 400 MHz spectrometer using CDCl_3_ as the solvent and the spectra were referenced to residual chloroform (*δ* 7.26 ppm). Mass spectra were recorded on an ESI-TOF mass spectrometer.

The aldehyde **5**, **Lipid** **14**, and **Lipid** **15** were synthesized according to our previously reported procedures. ^[1, 2]^

**Synthesis of Lipid 24:**

***Heptadecan-9-yl 8-bromooctanoate***

The 8-Bromooctanoic acid **1** (1.13 g, 5.08 mmol, 1.3 equiv.), Heptadecan-9-ol **2** (1.0 g, 3.90 mmol, 1 equiv.), EDC.HCl (1.50 g, 7.80 mmol, 2 equiv.) and DMAP (95 mg, 0.78 mmol, 20 mol%) were dissolved in dry CH_2_Cl_2_ (30 mL) under argon atmosphere and stirred for the overnight at room temperature. After that, the reaction was quenched with sat. NaHCO_3_ followed by extract with CH_2_Cl_2_ (3 times). Then the organic portion was washed with brine solution and dried over anhydrous Na_2_SO_4_. The solvent was evaporated and the residue was purified by column chromatography using 0-5% EtOAc in hexane to get the desired bromide **3** (1.3 g, 72%) as colorless liquid.

^1^H NMR (400 MHz, CDCl_3_): *δ* 4.86 (1 H, quint, *J* = 4.8 Hz), 3.52 (1 H, t, *J* = 6.8 Hz), 3.40 (1 H, t, *J* = 6.8 Hz), 2.28 (2 H, t, *J* = 7.6 Hz), 1.85 (1 H, quint, *J* = 7.6 Hz), 1.77 (1 H, quint, *J* = 7.6 Hz), 1.71-1.38 (10 H, m), 1.38-1.14 (26 H, m), 0.88 (6 H, t, *J* = 7.2 Hz).

***Heptadecan-9-yl 8-((2-hydroxyethyl)amino)octanoate***

The above bromide **3** (1.26 g, 2.73 mmol, 1 equiv.), ethanolamine (4.95 mL, 82 mmol, 30 equiv.) were dissolved in ethanol (5 mL) and stirred for the overnight at 65 ºC. After that, the solvent was evaporated under reduced pressure, then reaction mixture poured in the water and extracted with ethyl acetate (3 times). The organic portion was washed with brine solution and dried over with anhydrous Na_2_SO_4_. The solvent was evaporated and the residue was purified by column chromatography using 0-15% Methanol (1% triethylamine) in Chloroform to get the desired ethanolamine **4** (1.0 g, 83%) as colorless liquid.

^1^H NMR (400 MHz, CDCl_3_): *δ* 4.86 (1 H, quint, *J* = 6.4 Hz), 3.64 (2 H, t, *J* = 5.2 Hz), 2.78 (2 H, t, *J* = 5.2 Hz), 2.62 (2 H, t, *J* = 7.2 Hz), 2.28 (2 H, t, *J* = 7.6 Hz), 1.69-1.56 (4 m, H), 1.55-1.42 (8 H, m), 1.40-1.16 (26 H, m), 0.88 (6 H, t, *J* = 7.2 Hz).

ESI-MS: *m/z* 442.7 [M+1]^+^

***8-((8-(Heptadecan-9-yloxy)-8-oxooctyl)(2-hydroxyethyl)amino)octyl 2-hexyldecanoate***

To the above ethanolamine **4** (340 mg, 0.77 mmol, 1 equiv.), a solution of 8-oxooctyl 2-hexyldecanoate **5** (353 mg, 0.92 mmol, 1.2 equiv.) in dry CH_2_Cl_2_ (20 mL) was added in under argon atmosphere and stirred for 2 hr at room temperature. Later, sodium triacetoxyborohydride (325 mg, 1.54 mmol, 2.0 equiv.) was added and stirred for the overnight at the same temperature. Then, the reaction was quenched with sat.NaHCO_3_ solution followed by extract with CH_2_Cl_2_ (3 times). The organic layer was washed with brine solution and dried over anhydrous Na_2_SO_4_. The solvent was evaporated on rotary evaporator and the residue was purified by column chromatography using 0-5% Isopropanol in CHCl_3_ to obtain the desired product **6** (600 mg, 92%) as colorless liquid.

^1^H NMR (400 MHz, CDCl_3_): *δ* 4.86 (1 H, quint, *J* = 6.4 Hz), 4.06 (2 H, t, *J* = 6.8 Hz), 3.60-3.43 (2 H, br), 2.67-2.53 (2 H, br), 2.52-2.36 (4 H, br), 2.36-2.31 (1 H, m), 2.27 (2 H, t, *J* = 7.6 Hz), 1.77-1.38 (22 H, m), 1.38-1.11 (52 H, m), 0.88 (12 H, t, *J* = 7.2 Hz).

ESI-MS: *m/z* 809.2 [M+1]^+^

***8-((2-((4-(Dimethylamino)butanoyl)oxy)ethyl)(8-(heptadecan-9-yloxy)-8-oxooctyl)amino)octyl 2-hexyldecanoate***

The above alcohol **6** (600 mg, 0.74 mmol, 1.0 equiv.), *N, N*-dimethyl aminobutyric acid hydrochloride (248 mg, 1.48 mmol, 2.0 equiv.), EDC.HCl (283 mg, 1.48 mmol, 2.0 equiv.) and DMAP (18 mg, 0.15 mmol, 20 mol%) were dissolved in dry CH_2_Cl_2_ (20 mL) under argon atmosphere and stirred for the overnight at room temperature. After that, the reaction was quenched with sat. NaHCO_3_ followed by extract with CH_2_Cl_2_ (3 times). Then the organic portion was washed with brine solution and dried over anhydrous Na_2_SO_4_. The solvent was evaporated and the residue was purified by column chromatography using 0-10% IPA in CHCl_3_ to bestow **Lipid 24** (540 mg, 79%) as colorless oil.

^1^H NMR (400 MHz, CDCl_3_): *δ* 4.86 (1 H, quint, *J* = 6.4 Hz), 4.11 (2 H, t, *J* = 6.4 Hz), 4.06 (2 H, t, *J* = 6.8 Hz), 2.67 (2 H, t, *J* = 6.4 Hz), 2.43 (4 H, t, *J* = 7.6 Hz), 2.37-2.24 (3 H, m), 2.34 (2 H, t, *J* = 7.6 Hz), 2.27 (2 H, t, *J* = 7.6 Hz), 2.24 (6 H, s), 1.80 (2 H, quint, *J* = 7.2 Hz), 1.74-1.54 (10 H, m), 1.54-1.46 (4 H, m), 1.46-1.36 (6 H, m), 1.36-1.11 (54 H, m), 0.88 (12 H, t, *J* = 7.2 Hz).

ESI-MS: *m/z* 922.2 [M+1]^+^; 462.0 [M/2+1]^+^

**Synthesis of Lipid 30:**

***8-((2-hydroxyethyl)amino)octyl 2-hexyldecanoate***

To a solution of 8-oxooctyl 2-hexyldecanoate **5** (910 mg, 2.38 mmol, 1 equiv.) in dry CH_2_Cl_2_ (80 mL), ethanolamine (174 µl, 2.86 mmol, 1.2 equiv.) was added under argon atmosphere and stirred for 2 hr. at room temperature. Then sodium triacetoxyborohydride (1.0 g, 4.76 mmol, 2 equiv.) was added portion wise and stirred for overnight at the same temperature. After that, the reaction was quenched with sat.NaHCO_3_ solution followed by extract with CH_2_Cl_2_ (3 times). The organic layer was washed with brine solution and dried over anhydrous Na_2_SO_4_. The solvent was evaporated and the residue was purified by column chromatography using 0-10% MeOH in CHCl_3_ to get desired ethanolamine **7** (450 mg, 45%) as a colorless liquid.

^1^H NMR (400 MHz, CDCl_3_): *δ* 4.05 (2 H, t, *J* = 6.8 Hz), 3.77 (2 H, t, *J* = 1.8 Hz ), 2.92 (2 H, t, *J* = 5.2 Hz ), 2.76 (2 H, t, *J* = 7.6 Hz ), 2.36-2.24 (1 H, m), 1.71- 1.51 (6 H, m), 1.50-1.09 (30 H, m), 0.87 (6 H, t, *J* = 6.4 Hz).

ESI-MS: *m/z* 428.7 [M+1]^+^

***Undecyl 6-hydroxyhexanoate***

The 6-((tert-butyldimethylsilyl)oxy)hexanoic acid (4.0 g, 16.26 mmol, 1 equiv.), undecanol (4.04 mL, 19.51 mmol, 1.2 equiv.), EDC.HCl (4.66 g, 24.39 mmol, 1.5 equiv.) and DMAP (297 mg, 2.44 mmol, 15 mol%) were dissolved in dry CH_2_Cl_2_ (80 mL) under argon atmosphere and stirred for the overnight at room temperature. After that, the reaction was quenched with sat. NaHCO_3_ followed by extract with CH_2_Cl_2_ (3 times). Then the organic portion was washed with brine solution and dried over with anhydrous Na_2_SO_4_. The solvent was evaporated and the residue was purified by column chromatography using 0-5% EtOAc in hexane to obtain the desired silyl product (6.1 g, 94%) as a colorless liquid. After that, the silyl product (6.1 g, 15.25 mmol, 1 equiv.) was dissolved in THF (30 mL) and TBAF (23 mL, 1.0 M in THF, 22.87 mmol, 1.5 equiv.) was added, and stirred for 5 hr at room temperature. Then, the reaction was quenched with sat. NH_4_Cl solution and extracted with ethyl acetate (3 times), and the organic portion was washed with brine solution and dried over with anhydrous Na_2_SO_4_. The solvent was evaporated under reduced pressure and the residue purified by column chromatography using 0-20% EtOAc in hexane to obtain undecyl 6-hydroxyhexanoate **8** (4.0 g, 92%) as a colorless liquid.

^1^H NMR (400 MHz, CDCl_3_): *δ* 4.06 (2 H, t, *J* = 6.8 Hz), 3.65 (2 H, t, *J* = 5.6 Hz), 2.31 (2 H, t, *J* = 7.6 Hz), 1.74-1.51 (6 H, m), 1.47-1.17 (18 H, m), 0.88 (3 H, t, *J* = 6.8 Hz).

***Undecyl 6-oxohexanoate***

To a solution undecyl 6-hydroxyhexanoate **8** (1.0 g, 3.5 mmol, 1 equiv.) in dry CH_2_Cl_2_ (30 mL), molecular sieves (4Å MS) were added under argon atmosphere and then PCC (1.5 g, 7.0 mmol, 2 equiv.) was added portion wise and stirred for 2 hr. After that, the reaction mixture was filtered through silica gel column to remove PCC followed by wash with 20% ethyl acetate in hexane (2 × 100 mL). The solvent was evaporated under reduced pressure to obtain the undecyl 6-oxohexanoate **9** (0.85 g, 85%) as a colorless liquid.

^1^H NMR (400 MHz, CDCl_3_): *δ* 9.77 (1 H, t, *J* = 1.6 Hz), 4.06 (2 H, t, *J* = 6.8 Hz), 2.50-2.41 (2 H, m), 2.40-2.26 (2 H, m), 1.79-1.52 (6 H, m), 1.40-1.15 (16 H, m), 0.88 (3 H, t, *J* = 7.2 Hz).

***8-((2-Hydroxyethyl)(6-oxo-6-(undecyloxy)hexyl)amino)octyl 2-hexyldecanoate***

To the above ethanolamine **7** (220 mg, 0.51 mmol, 1 equiv.), a solution of undecyl 6-oxohexanoate **9** (190 mg, 0.67 mmol, 1.2 equiv.) in dry CH_2_Cl_2_ (15 mL) was added in under argon atmosphere and stirred for 2 hr at room temperature. Later sodium triacetoxyborohydride (217 mg, 1.03 mmol, 2.0 equiv.) was added and stirred for the overnight at the same temperature. Then the reaction was quenched with sat.NaHCO_3_ solution followed by extract with CH_2_Cl_2_ (3 times). The organic layer was washed with brine solution and dried over anhydrous Na_2_SO_4_. The solvent was evaporated on rotary evaporator and the residue was purified by column chromatography using 0-5% Isopropanol in CHCl_3_ to obtain the desired lipid **10** (270 mg, 75%) as colorless liquid.

^1^H NMR (400 MHz, CDCl_3_): *δ* 4.06 (2 H, t, *J* = 6.8 Hz), 4.05 (2 H, t, *J* = 6.8 Hz), 3.52 (2 H, t, *J* = 5.2 Hz ), 2.57 (2 H, t, *J* = 5.2 Hz ), 2.44 (4 H, q, *J* = 6.4 Hz ), 2.36-2.24 (1 H, m), 2.30 (2 H, t, *J* = 7.6 Hz), 1.71- 1.50 (12 H, m), 1.50-1.37 (8 H, m), 1.37-1.13 (40 H, m), 0.95-0.81 (9 H, m).

ESI-MS: *m/z* 697.0 [M+1]^+^

***8-((2-((4-(Dimethylamino)butanoyl)oxy)ethyl)(6-oxo-6-(undecyloxy)hexyl)amino)octyl 2-hexyldecanoate***

The above alcohol **10** (265 mg, 0.38 mmol, 1 equiv.), *N, N*-dimethyl aminobutyric acid hydrochloride (127 mg, 0.76 mmol, 2 equiv.), EDC.HCl (145 mg, 0.76 mmol, 2 equiv.) and DMAP (10 mg, 0.08 mmol, 20 mol%) were dissolved in dry CH_2_Cl_2_ (15 mL) under argon atmosphere and stirred for 24 hr at room temperature. After that, the reaction was quenched with sat. NaHCO_3_ followed by extract with CH_2_Cl_2_ (3 times). Then the organic portion was washed with brine solution and dried over anhydrous Na_2_SO_4_. The solvent was evaporated and the residue was purified by column chromatography using 0-10% Isopropanol in CHCl_3_ to obtain **Lipid 30** (250 mg, 82%) as pale yellowish liquid.

^1^H NMR (400 MHz, CDCl_3_): *δ* 4.10 (2 H, t, *J* = 6.4 Hz), 4.06 (2 H, t, *J* = 6.8 Hz), 4.05 (2 H, t, *J* = 6.8 Hz ), 2.67 (2 H, t, *J* = 6.4 Hz ), 2.49-2.39 (4 H, m ), 2.37-2.26 (1 H, m), 2.34 (2 H, t, *J* = 7.6 Hz), 2.29 (4 H, t, *J* = 7.6 Hz), 2.22 (6 H, s), 1.79 (2 H, quint, *J* = 7.6 Hz), 1.70- 1.52 (12 H, m), 1.51-1.37 (8 H, m), 1.37-1.17 (40 H, m), 0.93-0.81 (9 H, m).

ESI-MS: *m/z* 810.1 [M+1]^+^; 405.9 [M/2+1]^+^

**Synthesis of Lipid 31:**

***((2-Hydroxyethyl) azanediyl) bis(octane-8,1-diyl) bis(2-hexyldecanoate)***

To a solution of 8-oxooctyl 2-hexyldecanoate **5** (760 mg, 2.0 mmol, 1 equiv.) in dry CH_2_Cl_2_ (20 mL), Ethanolamine (60 µL, 1.0 mmol, 0.5 equiv.) was added under argon atmosphere and stirred for 2 hr. at room temperature. After that, sodium triacetoxyborohydride (630 mg, 3.0 mmol, 1.5 equiv.) was added portion wise and left for the overnight stirring at the same temperature. Then, the reaction was quenched with sat. NaHCO_3_ and followed by extract with CH_2_Cl_2_ (3 times). The organic portion was washed with brine solution and dried over anhydrous Na_2_SO_4_. The solvent was evaporated and the crude was purified by column chromatography using 0-6% Isopropanol in CHCl_3_ to get the desired ethanolamine **11** (620 mg, 80%) as colorless liquid.

^1^H NMR (400 MHz, CDCl_3_): *δ* 4.05 (4 H, t, *J* = 6.8 Hz), 3.54 (2 H, t, *J* = 5.6 Hz), 2.59 (2 H, t, *J* = 5.6 Hz), 2.46 (4 H, t, *J* = 7.6 Hz), 2.34-2.24 (2 H, m), 1.68-1.50 (8 H, m), 1.48-1.36 (8 H, m), 1.36-1.16 (56 H, m), 0.86 (12 H, t, *J* = 6.8 Hz).

ESI-MS: m/z 795.1 [M+1]^+^

***10-(8-((2-hexyldecanoyl) oxy) octyl)-2-methyl-6-oxo-7-oxa-2,5,10-triazaoctadecan-18-yl 2-hexyldecanoate***

To a stirred solution of above alcohol **11** (250 mg, 0.314 mmol, 1.0 equiv.) in dry THF (15 mL), CDI (204 mg, 1.26 mmol, 4.0 equiv.) was added under argon atmosphere and stirred for the overnight at room temperature. Then, N, N-dimethyl ethylene diamine (67 µL, 0.63 mmol, 2.0 equiv.) and DMAP (7 mg, 0.063 mmol, 20 mol%) were added and stirred for 16 h at room temperature. After that, the reaction was quenched with water followed by extracted with EtOAc (3x20 mL), and the organic portion was washed with brine solution and dried over with anhydrous Na_2_SO_4_. The solvent was evaporated, and the residue was purified by column chromatography using 0-10% Isopropanol in CHCl_3_ to bestow **Lipid 31** (190 mg, 67%) as pale yellowish liquid.

^1^H NMR (400 MHz, CDCl_3_): *δ* 5.30 (1 H, brs), 4.15 (2 H, brs), 4.06 (4 H, t, *J* = 6.8 Hz), 3.27 (2 H, q, *J* = 5.2 Hz), 2.74 (2 H, brs), 2.57-2.39 (6 H, m), 2.35-2.28 (4 H, m), 2.26 (6 H, s), 1.63-1.53 (10 H, m), 1.51-1.37 (10 H, m), 1.36-1.24 (50 H, m), 0.87 (12 H, t, *J* = 6.8 Hz). ESI-MS: m/z 909.2 [M+1] ^+^; 455.6 [M/2+1] ^+^.


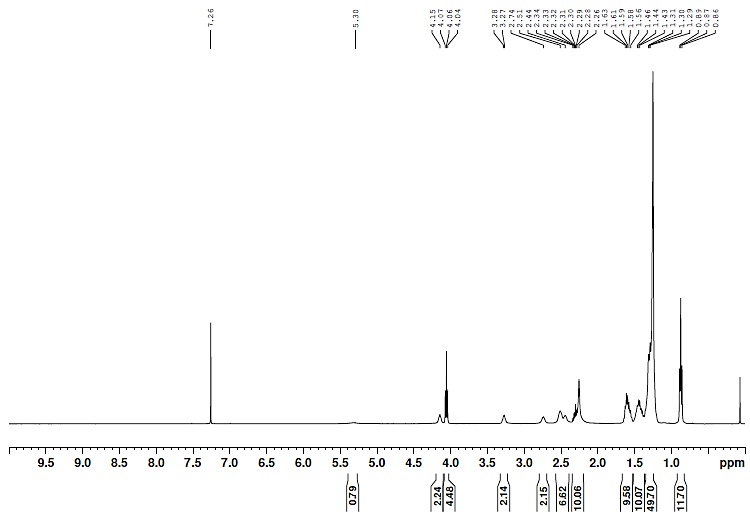


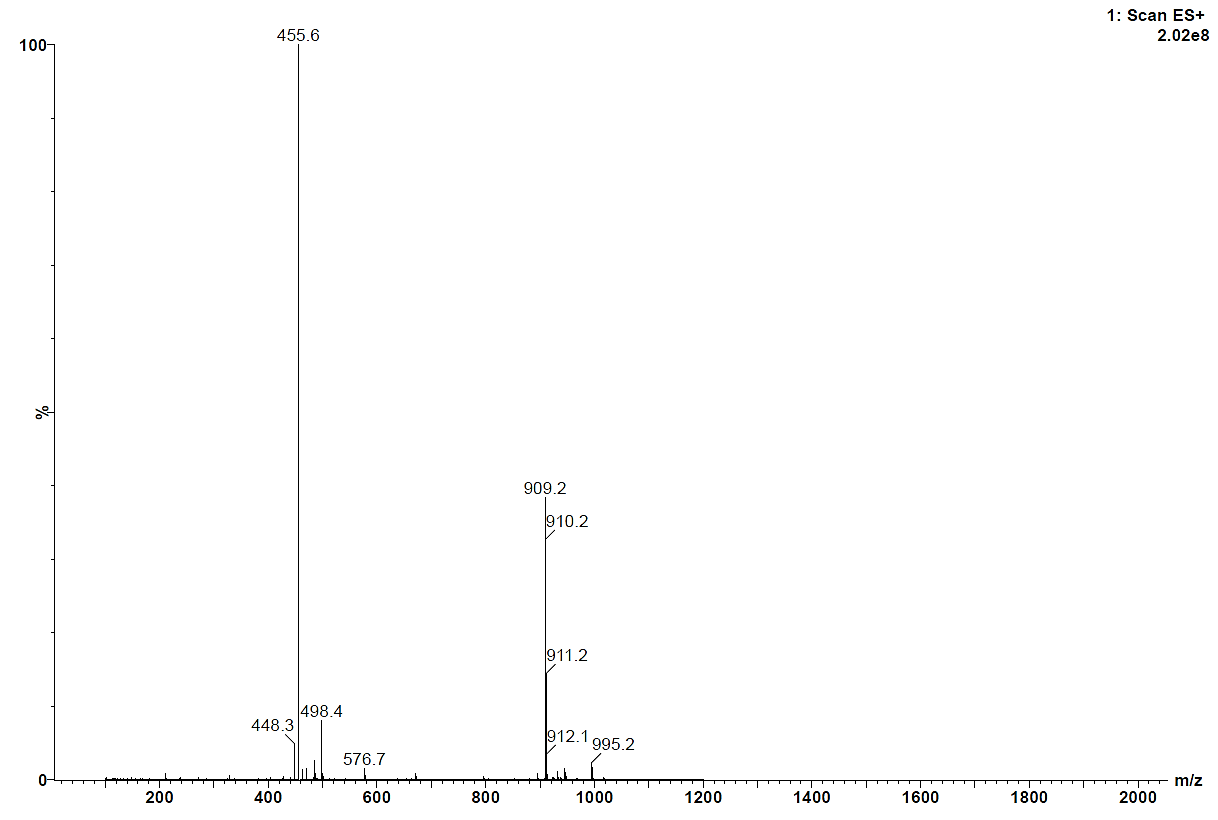

**Figure S1.** **YAP1 as a positive control for gene editing.** Percentage of gene editing events upon (Cas9 protein and sgYAP1) RNP complex transfection in FADU, and UMSCC-104 HNSCC cell lines. Data are means ±SD of three independent experiments.

**A**

**
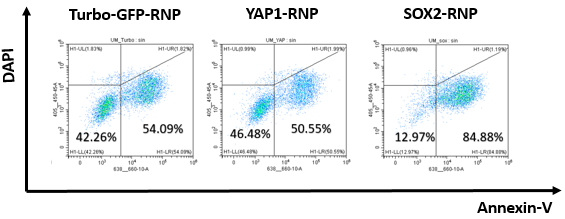
**

**B**

**Figure S2:** **DAPI-Annexin viability assay.** A) DAPI/annexin V assay of UMSCC cells treated with mock, sgTurboGFP, sgYAP1, and sgSox2 -RNP for 72 hours. B) Cell viability percentages were normalized to cells treated with sgTurbo-GFP-RNP. Data are means ±SD of two independent experiments. Unpaired T-test was used to assess the significance. P<0.05.

**A**

**
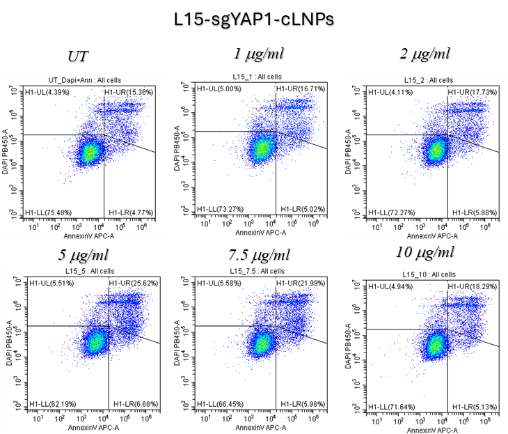

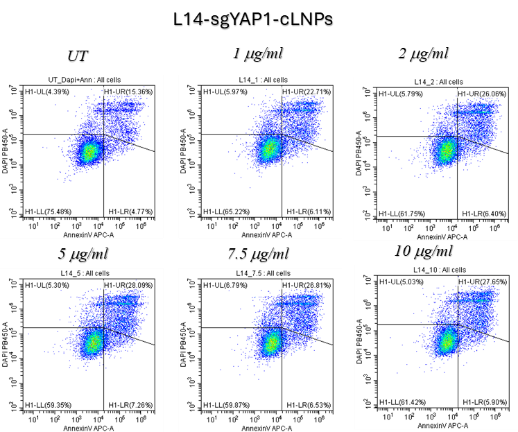
**

**
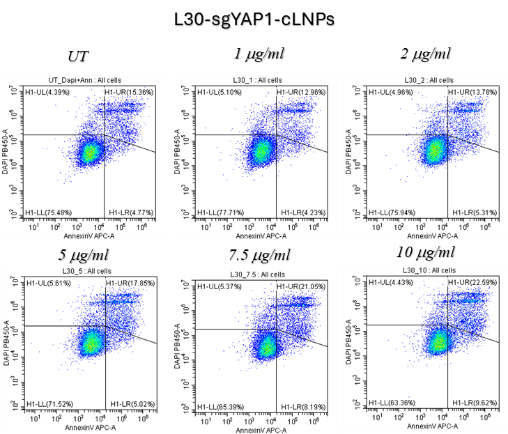

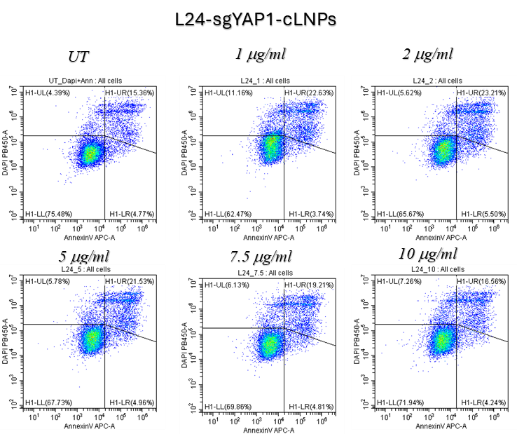
**

**
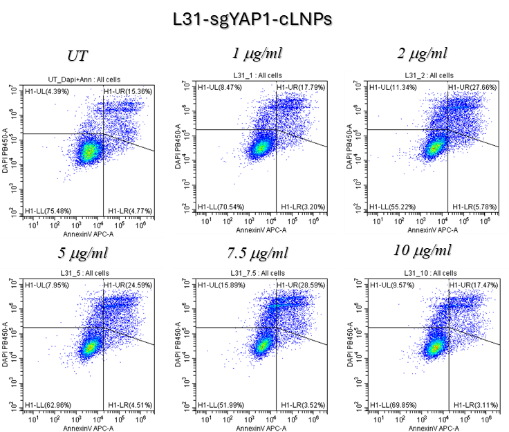
**

**B**

**C**

**Figure S3: Toxicity of CRISPR- LNPs.** A) Percentage of UMSCC cell viability 72 h post-transfection with either PBS or different concentrations of sgYAP1-cLNP (1–10 μg/mL of total RNA) as measured by DAPI-Annexin assay. B) Cell viability percentages were normalized to untreated cells. Data are means +SD of three independent experiments. C) YAP1 gene editing percentage of each formulation (10ug/ml), data are means +SD of three independent experiments.


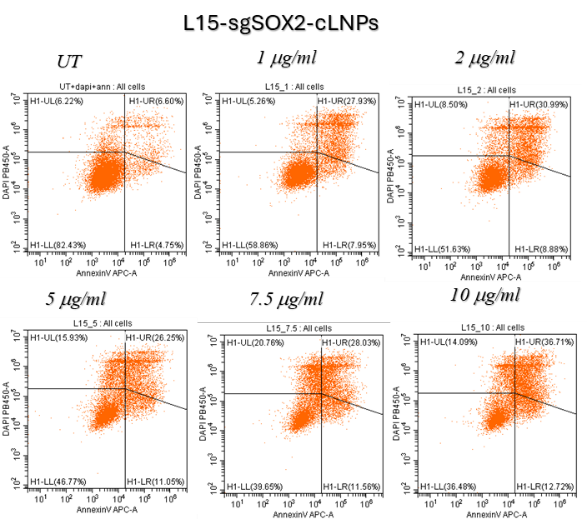

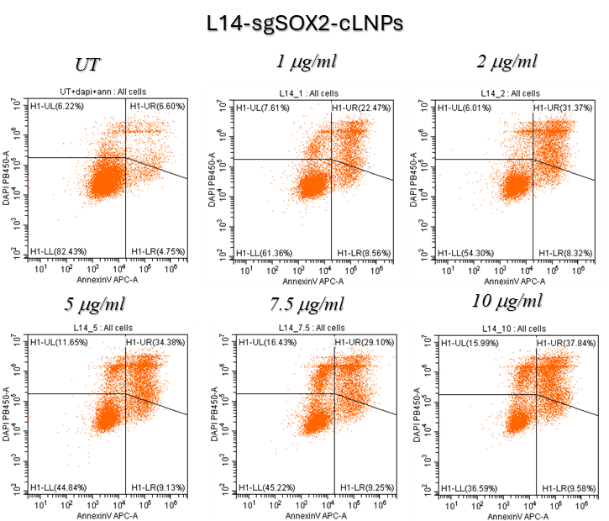


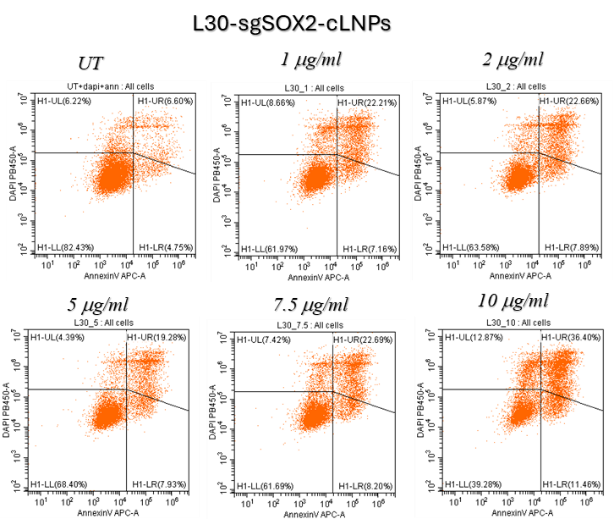

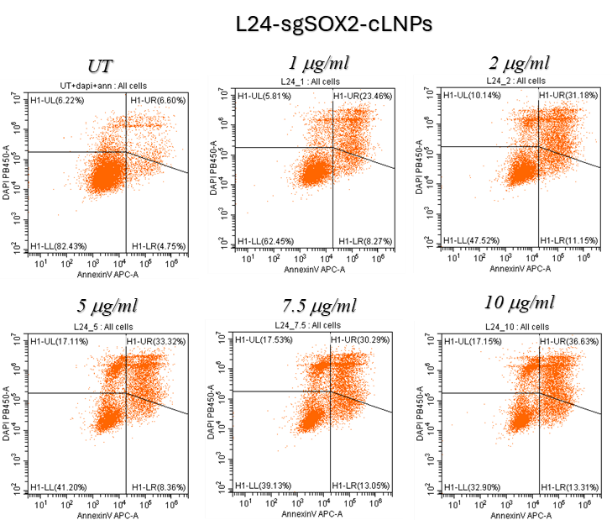


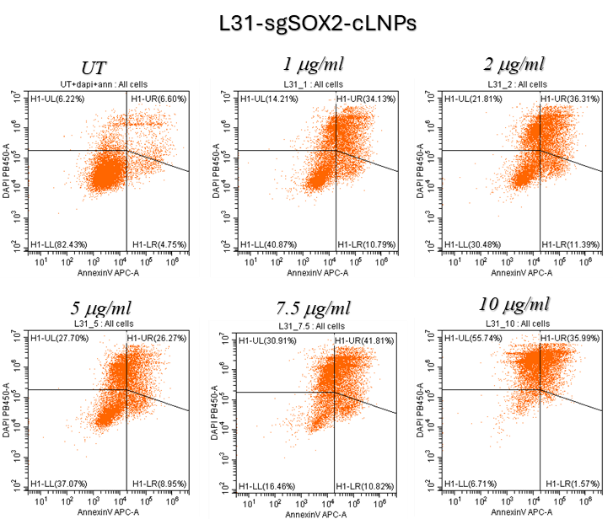


**Figure S4. Efficacy of CRISPR-LNPs.**  Representative dot plots of the percentage of UMSCC-104 cell viability 72h post-transfection with either PBS or different concentrations of sgSOX2-cLNP (1–10 μg/mL of total RNA) as measured by DAPI-Annexin assay.


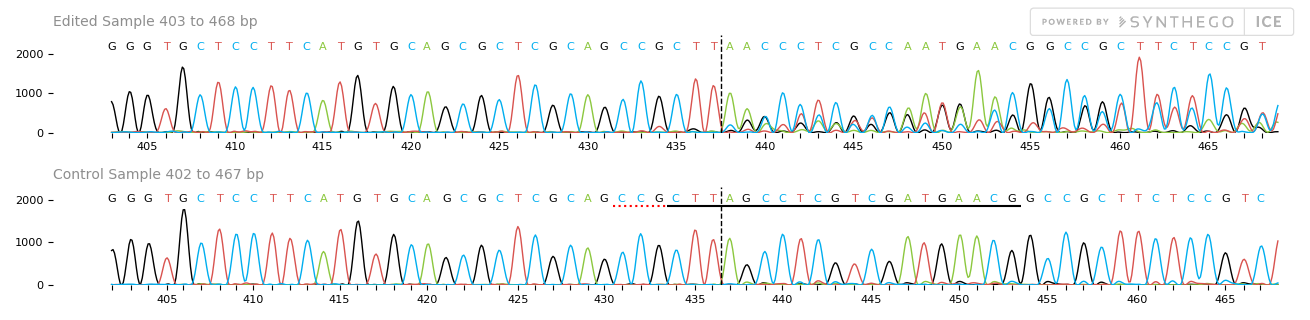
**A**

**
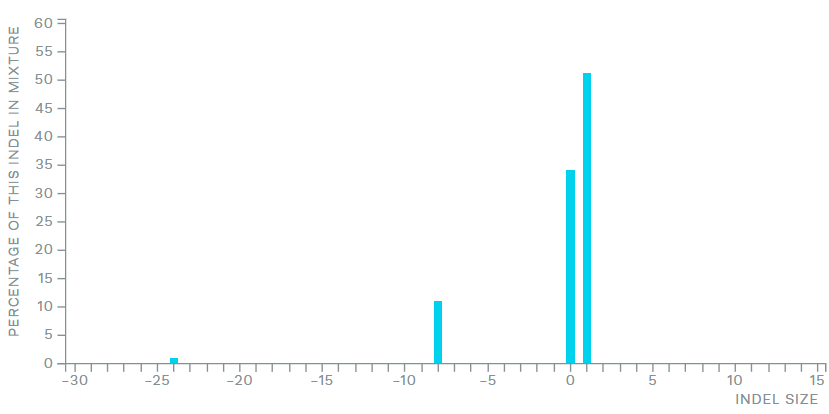
B**

**Figure S5: In vitro gene editing by CRISPR LNPs.** A) trace of an edited (top) and a control sample (bottom) in the area around the sgRNA (black line) and the PAM site (red, dotted line). The vertical black dotted line represents the double-strand break (DSB) site. Repair by NHEJ results in insertion after the DSB. B) Indel plot shows the Indel distribution in the edited population of genomes.


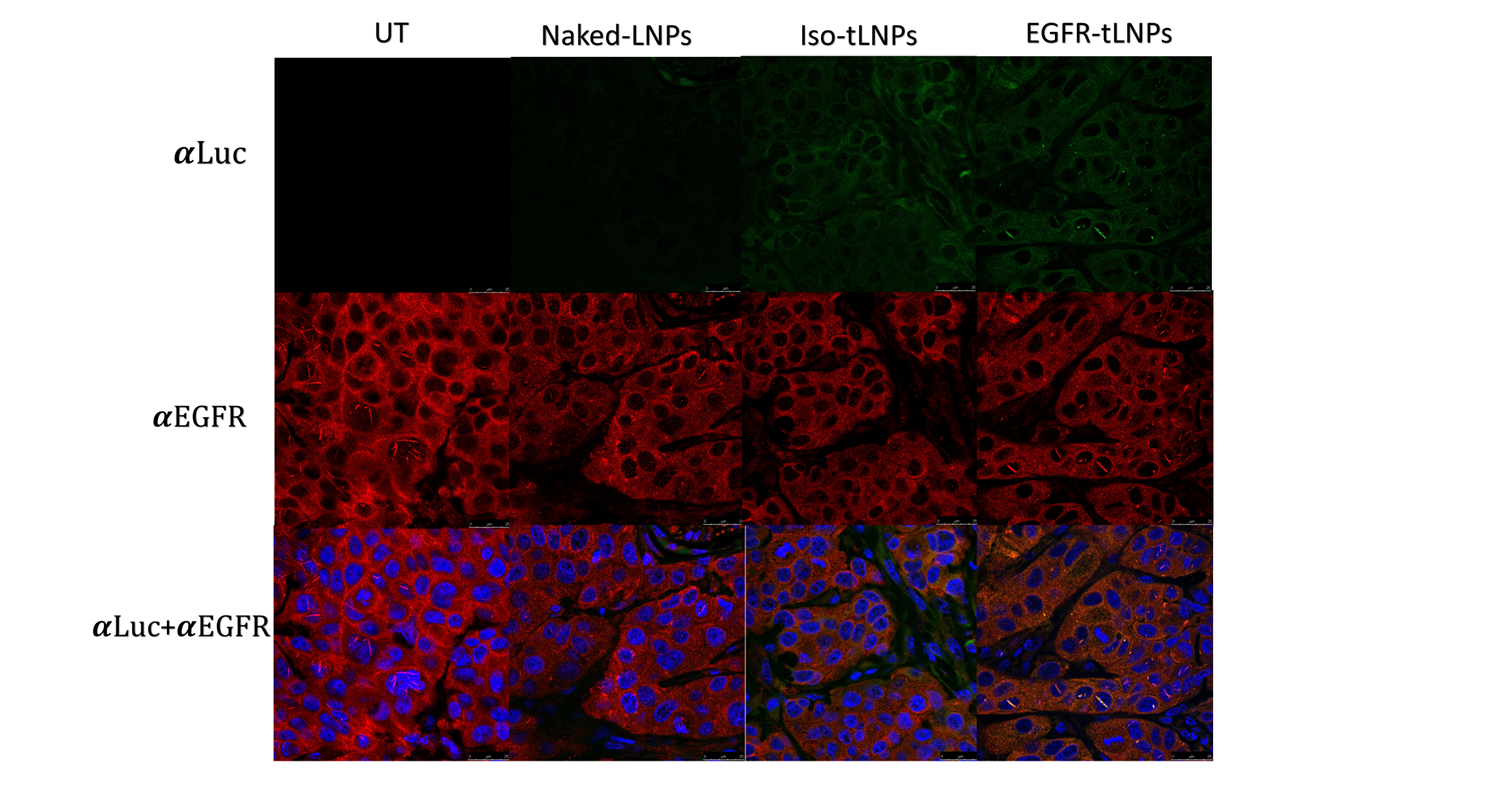


**A**

**B**

**Figure S6. Targeted and nontargeted L31-LNP encapsulating Luciferase-mRNA-delivery to HNSCC cells in a xerographic mouse model.** A) Representative tumor section of untreated (UT), naked, Iso- and EGFR-LNPs- treated mouse. Each section was stained with anti-luciferase (representative of luciferase transfection, Green stained), anti-EGFR (representative of tumor cells, Red stained), and section with both stains (representative of tumor cells transfected with luciferase, yellow stained). B) quantitative presentation of mean stain intensity of yellow (indicative of tumor cells transfected with luciferase LNPs), n=3/group. Data are presented as mean ±SD; One-way analysis of variance (ANOVA) with Tukey multiple comparison test was used to assess the significance. *p<0.05, **p<0.01, ***p<0.001.


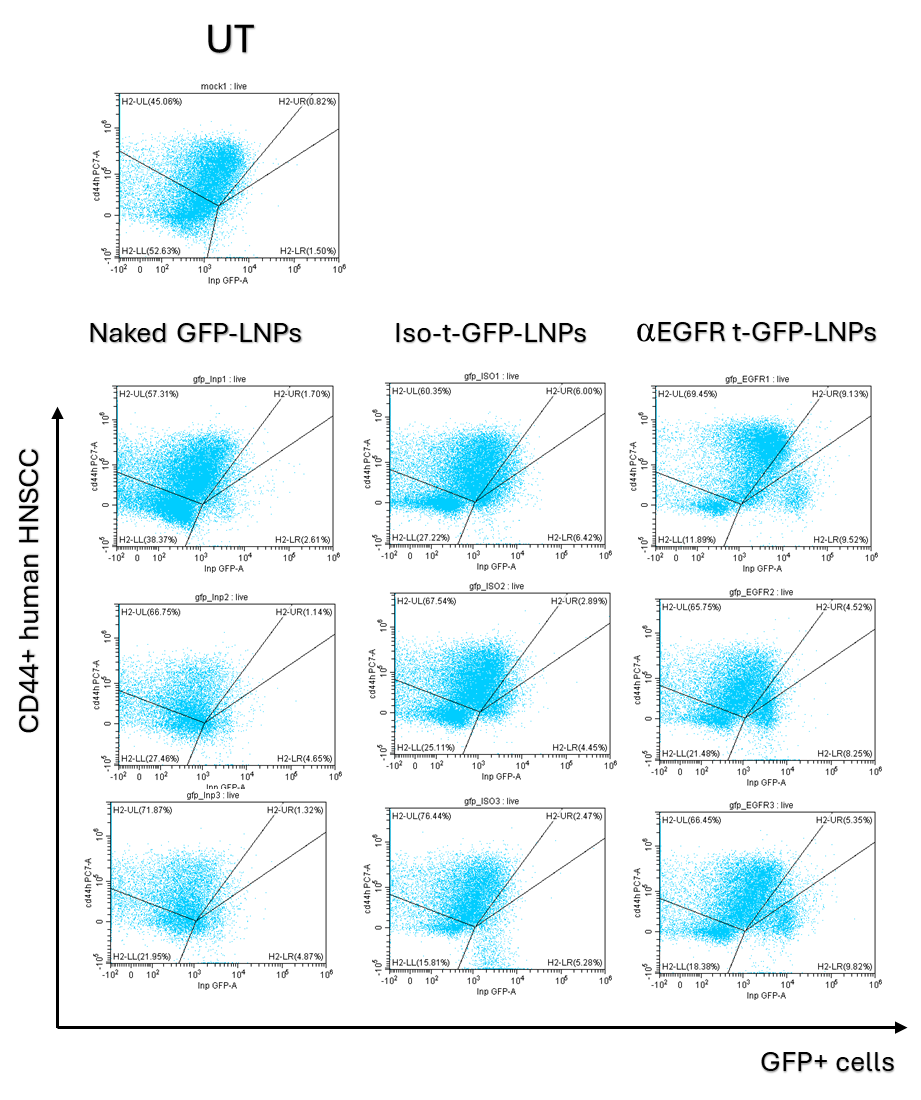


**Figure S7.** **GFP-Expression of targeted and non-targeted LNPs by CD44+ tumor cell in vivo.** A. Flow cytometry for human CD44+ HNSCC cells injected with either naked, iso- and αEGFR-GFP-LNPs compared to untreated HNSCC-bearing mice, each plot represents single cells from mouse stained with Cy7-PE-anti-human CD44 and GFP, n=3/group.

**
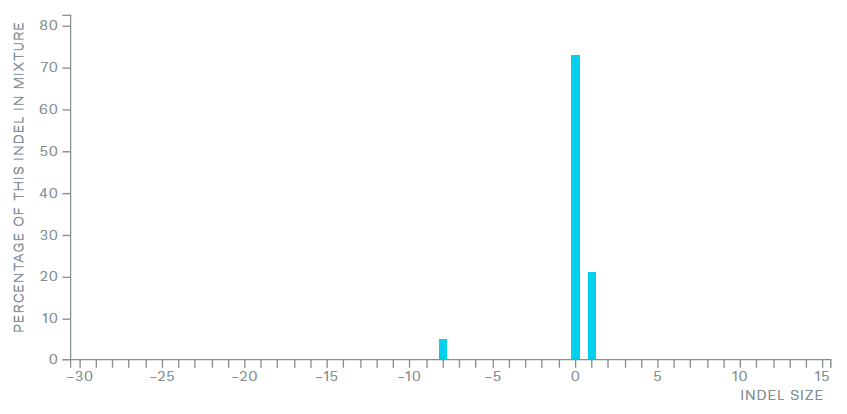
**

**Figure S8: In vivo gene editing by CRISPR LNPs in tumors**. Indel plot represents the T-SOX2-cLNP indel distribution in the edited population of genomes.

**A**


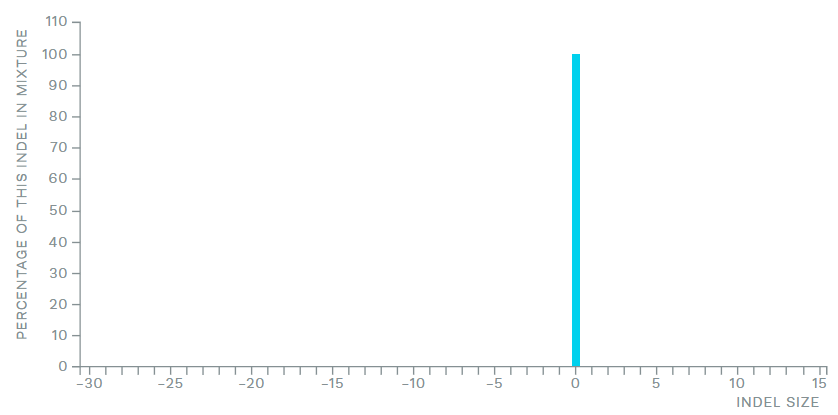


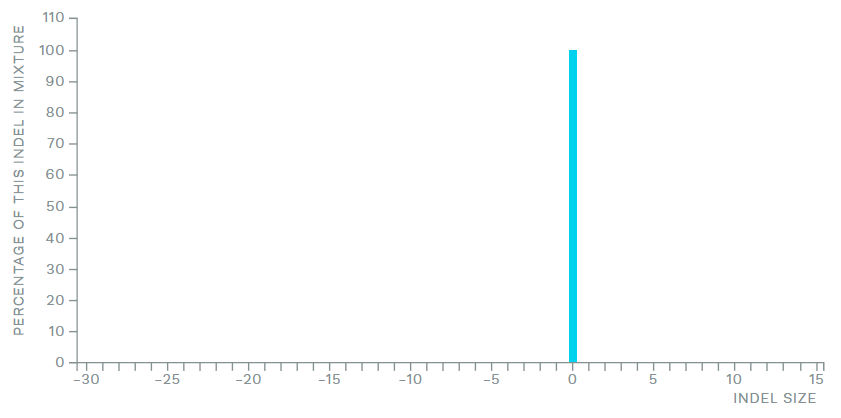


**B**

**Figure S9. In vivo gene editing by CRISPR LNPs in liver and spleen.** A) Indel plot represents the SOX2-cLNP indel distribution in the edited population of genomes in the liver. B) Indel plot represents the SOX2-cLNP indel distribution in the edited population of genomes in the spleen.

**
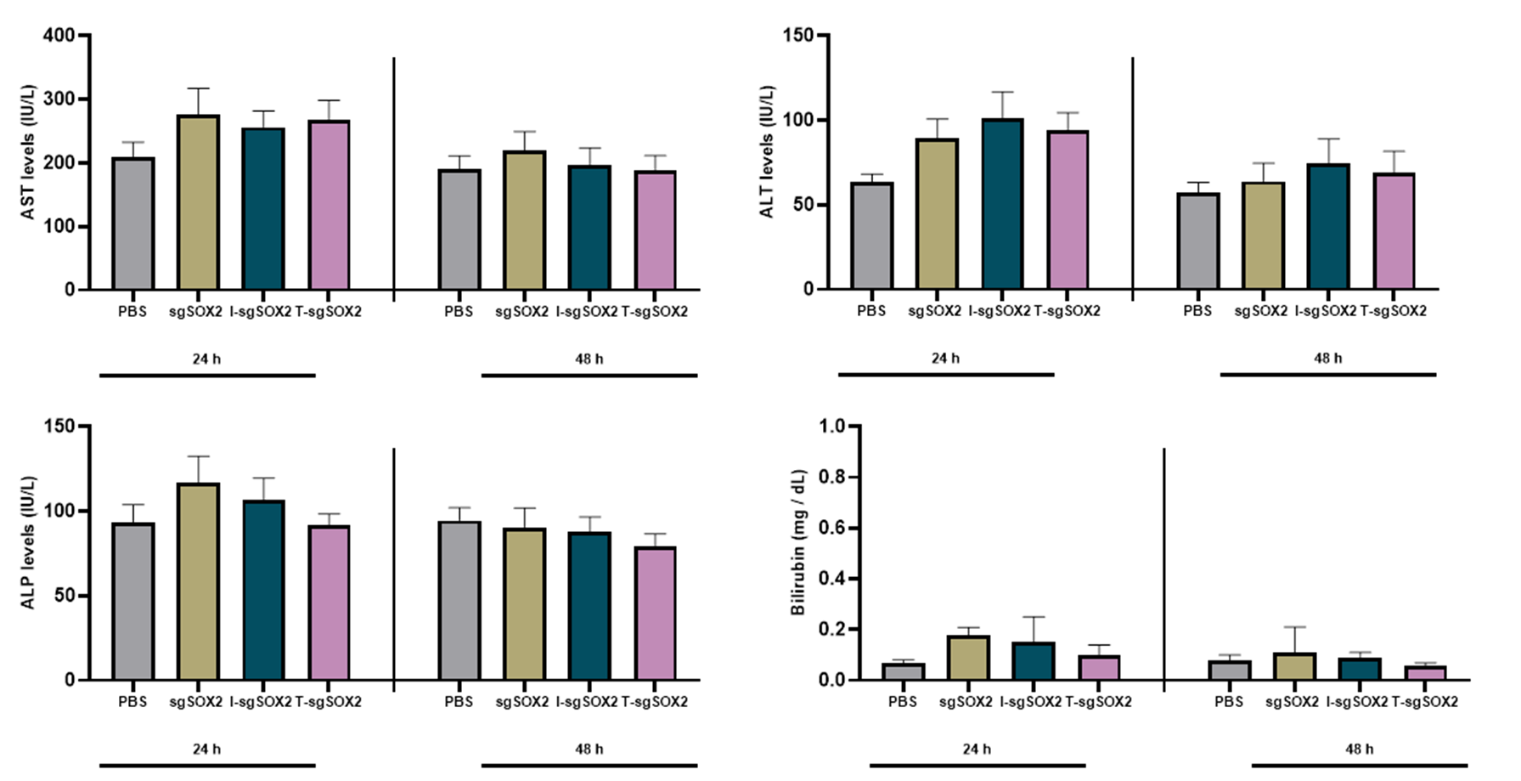
**

**A**

**
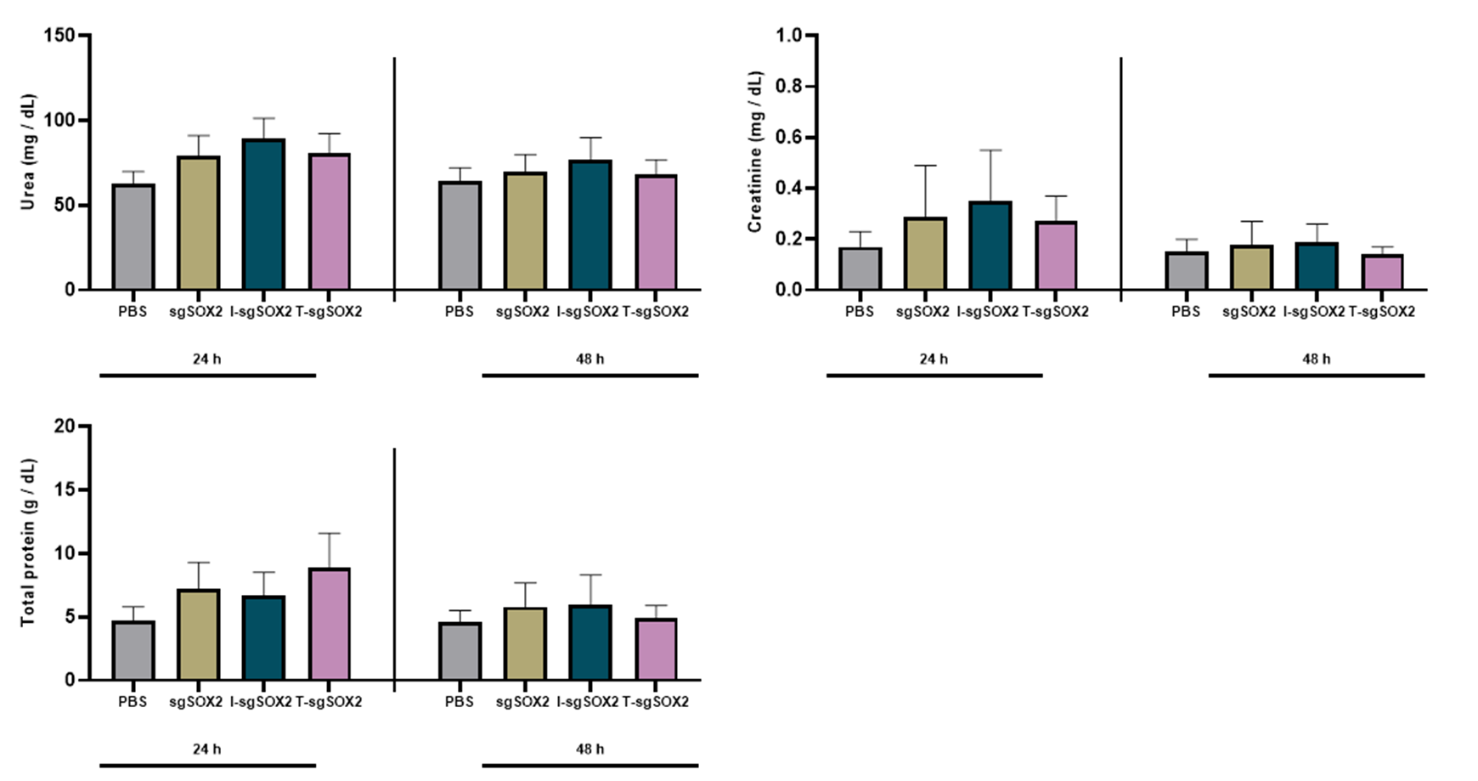
**

**B**

**C**

**
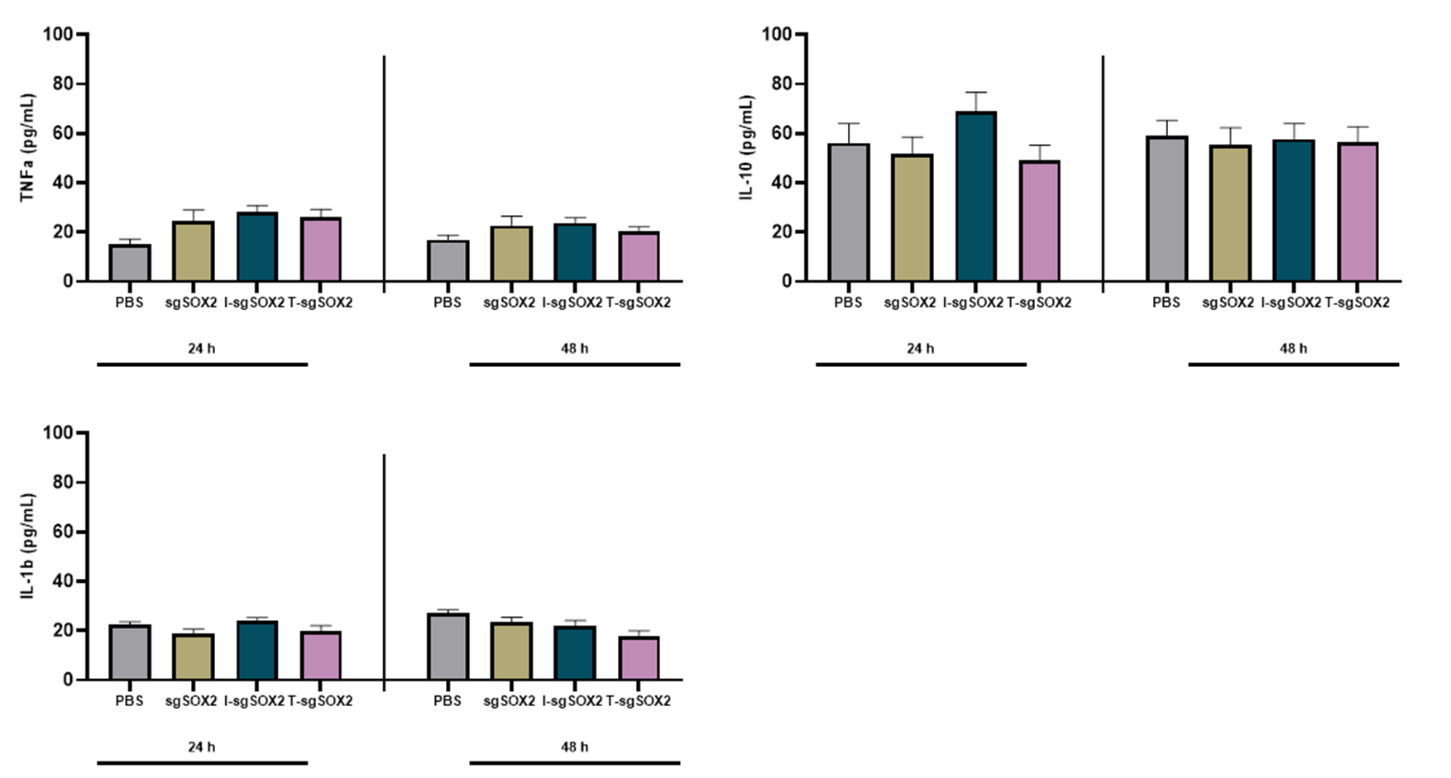
**

**Figure S10: Liver toxicity and Immunogenicity evaluation following intravenous injection of CRISPR-LNPs (cLNP).** Nude mice were injected intravenously with either EGFR-sgSOX2-cLNPs, Iso-sgSOX2-cLNPs, naked sgSOX2-cLNPs, and PBS at a dosage of 1 mg/Kg. The toxic effects of the sgSOX2-cLNPs were measured 24h and 48h post-injection. A) Liver enzymes elevation in the blood (alanine transaminase (ALT), aspartate aminotransferase (AST), and alkaline phosphatase (ALP)) and Bilirubin were evaluated 24 h and 48h post-injection. B) Kidney function-related parameters (urea, creatinine, and total protein) in the serum were evaluated 24 h and 48h post-injection. C) Immunogenicity was evaluated by measuring the serum levels of both pro and anti-inflammatory cytokines (IL-1β, TNF-α, and IL-10). Data are expressed as means+SD, with n=4 mice/group at each time-point; p= nonsignificant.


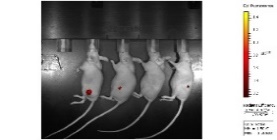

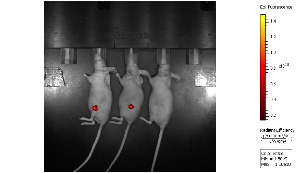

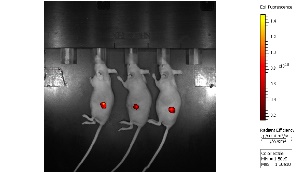


**W7**

**W8**

I-sgSOX2

T-sgSOX2

**W9**

**W10**

**W11**

**W12**


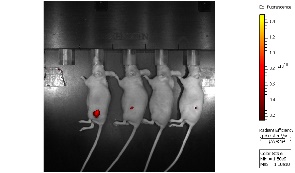

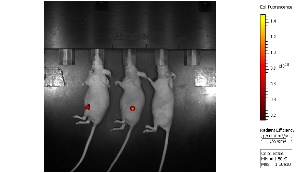

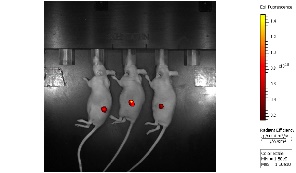

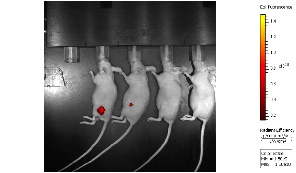

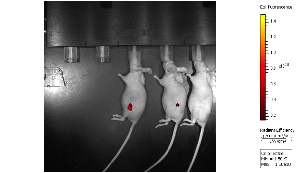

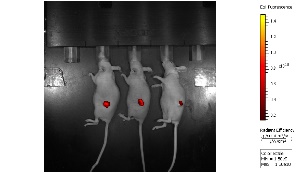

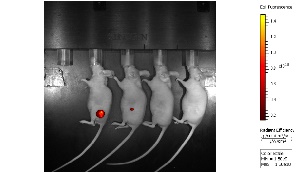

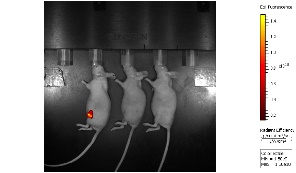

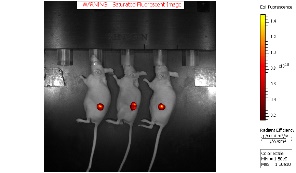

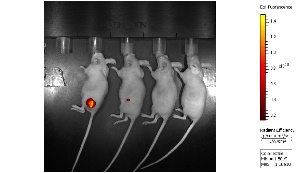

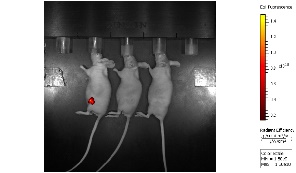

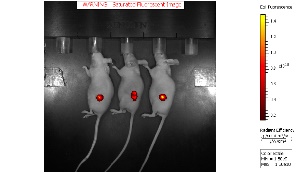

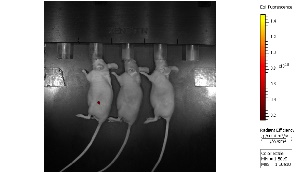

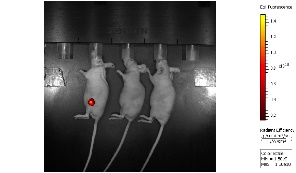

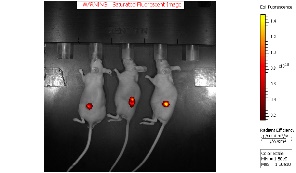

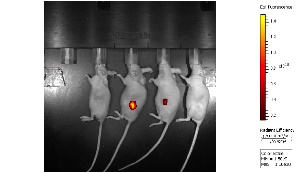

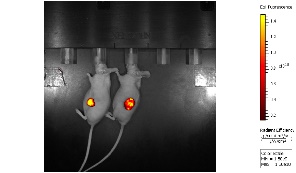

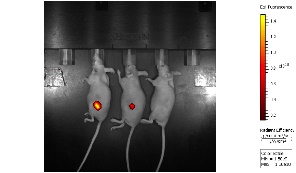

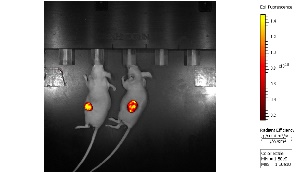

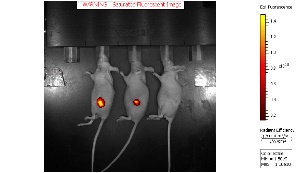


**Figure S11: Long-term follow-up.** Fluorescence imaging of UMSCC-bearing mice for a 12-week follow-up after 𝜶EGFR-T-SOX2-LNPs (T-sgSOX2) and Iso-I-SOX2-LNPs (I-sgSOX2) treatment.

**Figure S12: Tumor suppression rate relative to untreated (100%, unsuppressed growth) at week 6 of follow-up**. Data are means +SD of each mice group at week 6; one-way ANOVA with Tukey multiple comparison test was used to assess the significance. *p<0.05, ***p<0.001.

**References:**

[1] Ramishetti, S.; Hazan-Halevy, I.; Palakuri, R.; Chatterjee, S.; Naidu Gonna, S.; Dammes, Nanoparticles for RNA Delivery to Leukocytes. *Adv Mater* **2020**, *32* (12), e1906128. <https://doi.org/10.1002/adma.201906128>.

[2] Elia, U.; Ramishetti, S.; Rosenfeld, R.; Dammes, N.; Bar-Haim, E.; Naidu, G. S.; Makdasi, E.; Yahalom-Ronen, Y.; Tamir, H.; Paran, N.; Cohen, O.; Peer, D. Design of SARS-CoV-2 hFc-Conjugated Receptor-Binding Domain mRNA Vaccine Delivered via Lipid Nanoparticles. *ACS Nano* **2021**, *15* (6), 9627–9637. <https://doi.org/10.1021/acsnano.0c10180>.
